# Inhibition of HIV-1 infection in humanized mice and metabolic stability of protein phosphatase-1-targeting small molecule 1E7-03

**DOI:** 10.18632/oncotarget.19999

**Published:** 2017-08-07

**Authors:** Xionghao Lin, Namita Kumari, Catherine DeMarino, Yasemin Saygideğer Kont, Tatiana Ammosova, Amol Kulkarni, Marina Jerebtsova, Guelaguetza Vazquez-Meves, Andrey Ivanov, Kovalskyy Dmytro, Aykut Üren, Fatah Kashanchi, Sergei Nekhai

**Affiliations:** ^1^ Center for Sickle Cell Disease, College of Medicine, Howard University, Washington, DC, USA; ^2^ Department of Medicine, College of Medicine, Howard University, Washington, DC, USA; ^3^ Department of Microbiology, College of Medicine, Howard University, Washington, DC, USA; ^4^ Department of Pharmaceutical Sciences, College of Pharmacy, Howard University, Washington, DC, USA; ^5^ Lombardi Comprehensive Cancer Center, Georgetown University, Washington, DC, USA; ^6^ Laboratory of Molecular Virology, George Mason University, Manassas, VA, USA; ^7^ Department of Biochemistry, University of Texas Health Science Center, San Antonio, TX, USA; ^8^ Yakut Science Center for Complex Medical Problems, Yakutsk, Russia

**Keywords:** HIV-1, protein phosphatase-1, small molecule HIV-1 inhibitor, HIV-1 infected humanized mice, metabolic stability

## Abstract

We recently identified the protein phosphatase-1 - targeting compound, 1E7-03 which inhibited HIV-1 *in vitro*. Here, we investigated the effect of 1E7-03 on HIV-1 infection *in vivo* by analyzing its metabolic stability and antiviral activity of 1E7-03 and its metabolites in HIV-1 infected NSG-humanized mice. 1E7-03 was degraded in serum and formed two major degradation products, DP1 and DP3, which bound protein phosphatase-1 *in vitro*. However, their anti-viral activities were significantly reduced due to inefficient cell permeability. In cultured cells, 1E7-03 reduced expression of several protein phosphatase-1 regulatory subunits including Sds22 as determined by a label free quantitative proteomics analysis. In HIV-1-infected humanized mice, 1E7-03 significantly reduced plasma HIV-1 RNA levels, similar to the previously described HIV-1 transcription inhibitor F07#13. We synthesized a DP1 analog, DP1-07 with a truncated side chain, which showed improved cell permeability and longer pharmacokinetic retention in mice. But DP1-07 was less efficient than 1E7-03 as a HIV-1 inhibitor both *in vitro* and *in vivo*, indicating that the full side chain of 1E7-03 was essential for its anti-HIV activity. Analysis of 1E7-03 stability in plasma and liver microsomes showed that the compound was stable in human, primate and ferret plasma but not in rodent plasma. However, 1E7-03 was not stable in human liver microsomes. Our findings suggest that 1E7-03 is a good candidate for future development of HIV-1 transcription inhibitors. Further structural modification and advanced formulations are needed to improve its metabolic stability and enhance its antiviral activity in non-human primate animals and humans.

## INTRODUCTION

The eradication of Human Immunodeficiency Virus (HIV)-1 infection is challenging because of the HIV-1 integration and establishment of a latent infection. While selective combination anti-retroviral therapy (cART) efficiently suppresses ongoing HIV-1 replication, latent HIV-1 infection in stable reservoirs such as resting CD4+ T cells, naive T cells and CD34^+^ multipotent hematopoietic stem cells is not affected by the current antiretroviral drugs [1, 2]. Also, cART does not target HIV-1 transcription which is activated during viral reactivation in latent reservoirs. Therefore, targeting HIV-1 transcription with a novel anti-HIV drug may help in preventing HIV-1 reactivation and facilitate permanent HIV-1 suppression.

We had previously developed small molecule inhibitors of HIV-1 transcription that target host protein phosphatase-1 (PP1) [3, 4]. Our previous studies showed that HIV-1 Tat interaction with PP1 is critical for the HIV-1 transcription activation [5–7]. The initial hit compound, 1H4, and its cyclopentan quinoline derivative with improved activity, 1E7-03, bind non-competitively to PP1 *in vitro* without affecting PP1 enzymatic activity and prevent the interaction of HIV-1 Tat protein with PP1 [3, 4]. PP1 is dimer of a catalytic subunit (PP1α, PP1β/δ or PP1γ) and a regulatory subunit that targets PP1 holoenzyme into the specific cellular location and determines its activity and substrate specificity [8]. The interaction between PP1 catalytic and regulatory subunits occurs through a combination of short binding motifs, including an RVxF motif that is present in the majority of PP1 regulatory subunits [9]. The initial 1H4 compound was selected from a library of small molecules designed to bind to the PP1 RVxF binding site [4]. The 1E7-03 compound was selected from a library of 1H4 homologues which were also designed to fit PP1 RVxF binding cavity [3]. We recently showed that in addition to HIV-1, 1E7-03 also inhibited Ebola virus [10] and Rift valley fever virus [11] in infected cell cultures. While *in vitro* studies have yielded valuable information on the antiviral activity of 1E7-03 in cell cultures, the effect of 1E7-03 *in vivo* has not been explored. Thus, in the current study, we tested 1E7-03 metabolic stability and pharmacokinetics and analyzed its anti-HIV activity *in vivo*.

We first analyzed 1E7-03 stability in mouse plasma *in vitro* and its pharmacokinetics in mice. The stability of 1E7-03 in cell culture media and buffers with different pH was also analyzed. We generated a comprehensive profile of 1E7-03 degradation products (DPs) using a combination of LC/FT-MS/MS analysis with full (FL), neutral loss (NL) and multiple reaction monitoring (MRM) scans. Two major identified DPs, DP1 and DP3, were synthesized (Supplementary Figure 1), and tested for HIV-1 inhibition in cell culture. Their binding affinity to PP1 *in vitro* was tested using surface plasmon resonance technique. The effects on HIV-1 transcription and gene expression were also evaluated and compared with those of 1E7-03. We also tested cellular permeability of 1E7-03, DP1 and DP3. To understand the effect of 1E7-03 on PP1 in cultured cells, we performed label free quantitative proteomics analysis of HIV-1 infected CEM T cells treated with 1E7-03 versus untreated control. To determine the anti-HIV efficacy of 1E7-03 *in vivo*, we employed humanized NSG-BLT mice infected with the dual tropic HIV-1 89.6 virus [12]. For a comparison, we used the F07#13 compound, a Tat peptide mimetic inhibitor that was previously shown to inhibit HIV-1 in this mouse model [13]. We generated an analog of DP1, DP1-07 and tested its activity *in vivo*. The identified labile sites of 1E7-03 and species variations in plasma and liver enzymes prompted us to further test its stability in plasma obtained from guinea pig, ferret, monkey and human, and liver microsomes from mouse and human. To our knowledge, this is the first *in vivo* study conducted on a cyclopentan quinoline based compound.

## RESULTS

### Pharmacokinetics of 1E7-03 in mice and its degradation kinetics in mouse plasma

**Figure 1:**
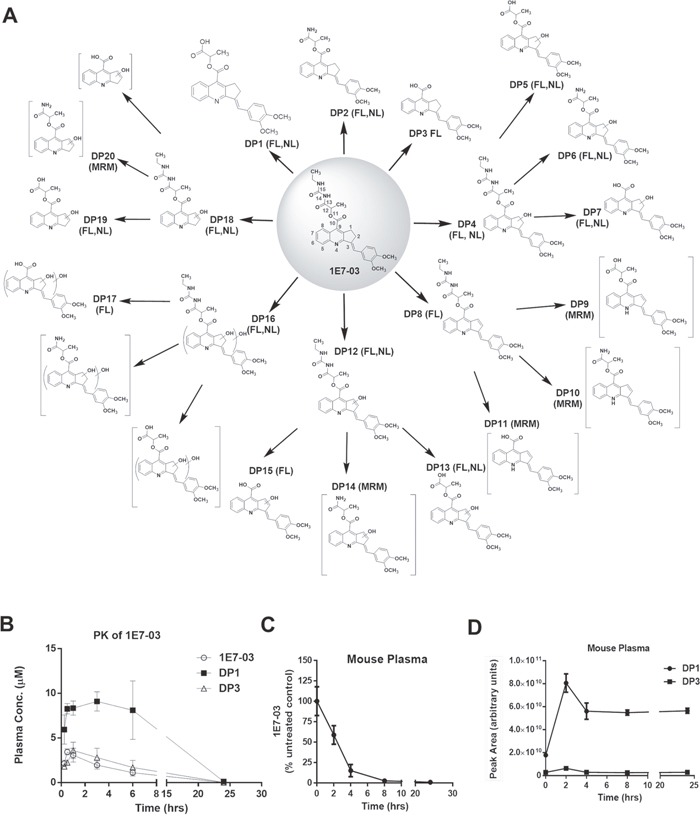
Degradation products of 1E7-03, pharmacokinetic of 1E7-03 in mice and its degradation kinetic in mouse plasma **(A)** All possible 1E7-03 degradation products (DPs) were identified when 1E7-03 was incubated under different experimental conditions, including mouse plasma (24 hrs) and buffers with pH 4, pH 7 and pH 10 (48 hrs) at 37°C. Small molecules were extracted with cold acetone precipitation and identified by LC/FT-MS analysis that included FL, NL and MRM scans as indicated on the figure. A total of 20 DPs are shown. Brackets indicate trace DPs that were only detected by MRM. Major DPs of 1E7-03 detected in mouse plasma were DP1, DP3, and followed by DP2 which was present at a very low level. **(B)** Pharmacokinetics of 1E7-03 in mice. Mice were injected i.p. with 30 mg/kg of 1E7-03. The concentrations of 1E7-03 and its major DPs were quantified by LC/FT-MS in the plasma at several time points for up to 24 hrs. Three mice were used for each time point. The means ± SD are shown. **(C-D)** Degradation kinetic of 1E7-03 in mouse plasma (C) and dynamic changes of the major DPs DP1 and DP3 (D). 1E7-03 (10 μM) was added to mouse plasma and incubated at 37°C for 24 hrs. Samples were collected at different time points, 1E7-03 and its DPs were extracted and quantified by LC/FT-MS analysis.

To analyze the metabolism of 1E7-03 *in vivo*, we performed pharmacokinetics (PK) in mice for 1E7-03. The compound was injected intraperitonealy (i.p.) and the time-dependent plasma concentrations of 1E7-03 and its major metabolites, DP1 and DP3 were measured (Figure 1A and 1B, see details for metabolites identification in Supplementary Materials, Supplementary Figures 2-7; Supplementary Tables 1 and 2). The main plasma pharmacokinetic parameters of 1E7-03 and its main metabolites are summarized in Table 1. The *T*_max_, *C*_max,_ *t*_1/2_ and *AUC*_last_ for 1E7-03 were found to be 0.5 hr, 3.43 μM, 3.39 hr and 12.22 μM·hr (Table 1). DP1 was accumulated with maximum at 3 hrs post injection (Figure 1B). To analyze 1E7-03 stability in mouse plasma, we incubated 10 μM 1E7-03 in the plasma at 37°C for 24 hrs. Samples were collected at different times, 1E7-03 and its DPs were extracted and quantified by LC/FT-MS analysis (see details for LC/FT-MS instrument and method validation in Supplemental Materials; Supplementary Figure 8 and Supplementary Tables 3-5). 1E7-03 degradation kinetics in plasma paralleled that in mice (Figure 1B-1D). The degradation dynamic plot showed that 40% of 1E7-03 had degraded after 2 hrs incubation and over 85% of 1E7-03 had degraded after 4 hrs of incubation (Figure 1C). The DP1 levels progressively increased with the degradation of 1E7-03 and reached their maximum at 2 hrs (Figure 1D). In contrast, DP3 was present at low levels, suggesting that DP1 remained the major degradation product. Thus, the 1E7-03 was not stable in mice and mouse plasma and its degradation was likely due to the hydrolysis of the amide bond C_13_–N_14_ (see central panel in Figure 1A).

**Table 1:** Pharmacokinetic parameters following intraperitoneal (i.p.) administration of compounds 1E7-03 and DP1-07 (mean, n=3)

Compounds	Dose (mg/kg)	*C*_max_ (μM)	*T*_max_ (hr)	*AUC*_last_ (hr μM)	*t*_1/2_ (hr)	*MRT*_last_ (hr)
1E7-03	30	3.43	0.5	12.22	3.39	2.40
DP1	-	9.09	3.0	123.97	3.20	4.98
DP3	-	3.61	1.0	30.66	2.44	4.39
DP1-07	100	42.71	6.0	705.61	14.29	10.68
DP1-07P1*	-	7.54	6.0	123.94	13.45	10.50
DP1-07P2*	-	23.71	6.0	288.38	4.66	8.86

Pharmacokinetic parameters were calculated by non-compartmental modeling using WinNonlin.

*C*_max_, peak plasma concentration; *T*_max_, time to peak plasma concentration; *AUC*_last_, area under the concentration vs. time curve from 0 hr up to the quantification time; *t*_1/2,_ elimination half-life; *MRT*_last_, mean resident time from 0 hr to the last detectable point. * The concentrations of compounds DP1-07P1 and DP1-07P2 in plasma were calculated by calibration curves of DP1 and DP3 due to lack of standards.

**Table 2:** Degradation products (DPs) of 1E7-03 characterized by LC/FT-MS after incubated in buffers^a^ and mouse plasma^b^ at 37°C

No.	*t*_R_ (min)	Formula	[M+H]^+^(*m/z*) measured	[M+H]^+^ (*m/z*) predicted	Error (ppm)	(+)-ESI-MS^n^ *m/z* (% of relative abundance)	Detected in				Scan modes
							A	N	B	S	
**DP1**	33.65	C_25_H_23_NO_6_	434.1622	434.1604	4.15	MS^2^ [434]: 362 (100)	+	++	+	+	FLNL (72 Da)
**DP2**	31.98	C_25_H_24_N_2_O_5_	433.1771	433.1763	1.85	MS^2^ [433]: 362 (100)	+	++	+	+	FLNL (71 Da)
**DP3**	27.93	C_22_H_19_NO_4_	362.1406	362.1392	3.87	MS^2^ [362]: 346 (100)	+	+	+	+	FL
**DP4**	30.73	C_28_H_29_N_3_O_7_	520.2107	520.2084	4.42	MS^2^ [520]: 502 (70), 475 (90), 449 (100)MS^3^ [520→475]: 378 (100)MS^3^ [520→449]: 378 (100)	+	++	+	–	FLNL (45, 71 Da)
**DP5**	29.58	C_25_H_23_NO_7_	450.1554	450.1553	0.22	MS^2^ [450]: 378 (60)	+	++	+	+	FLNL (72 Da)
**DP6**	28.40	C_25_H_24_N_2_O_6_	449.1694	449.1713	-4.23	MS^2^ [449]: 378 (100)	+	++	–	–	FLNL (71 Da)
**DP7**	24.84	C_22_H_19_NO_5_	378.1341	378.1341	0.00	MS^2^ [378]: 360 (10)	+	++	+	+	FLNL (18 Da)
**DP8^c^**	26.49	C_28_H_27_N_3_O_6_	502.1992	502.1978	2.79	MS^2^ [502]: 457 (80), 431 (100)MS^3^ [457]: 360 (100)MS^3^ [431]: 360 (100)	+	+	+	+	FL
**DP9**	30.71	C_25_H_21_NO_6_	360.1237	360.1236	0.28	*m/z* 432.14>360.12	–	––+	–	–	FLNLMRM
**DP10**	27.78	C_25_H_22_N_2_O_5_	360.1235	360.1236	-0.28	*m/z* 431.16>360.12	–	––+	–	–	FLNLMRM
**DP11**	25.72	C_22_H_17_NO_4_	360.1235	360.1236	-0.28	*m/z* 360.12>360.12	–	––+	–	–	FLNLMRM
**DP12**	29.35	C_28_H_27_N_3_O_7_	518.1950	518.1927	4.44	MS^2^ [518]: 473 (50), 447 (100)MS^3^ [518→447]: 376 (100)	+	++	–	–	FLNL (45, 71 Da)
**DP13**	28.64	C_25_H_21_NO_7_	448.1390	448.1396	-1.34	MS^2^ [448]: 376 (50)	+	++	–	+	FL,NL (72 Da)
**DP14**	27.20	C_25_H_22_N_2_O_6_	376.1193	376.1185	2.13	*m/z* 447.16>376.11	–	––+	–	–	FLNLMRM
**DP15**	27.13	C_22_H_17_NO_5_	376.1198	376.1185	3.46	MS^2^ [376]: 358 (20)	+	+	+	–	FL
**DP16**	29.35	C_28_H_29_N_3_O_8_	536.2057	536.2033	4.48	MS^2^ [536]: 491	+	++	–	–	FLNL (45 Da)
**DP17**	28.07	C_20_H_19_NO_6_	394.1308	394.1291	4.31	MS^2^ [394]: 376 (100), 332 (50)	–	+	+	–	FL
**DP18**	29.33	C_19_H_19_N_3_O_5_	370.1421	370.1430	-2.43	MS^2^ [370]: 325 (45), 299 (60), 228 (100)	+	++	–	–	FLNL (45, 71 Da)
**DP19**	28.6228.62	C_16_H_13_N_1_O_5_	300.0872228.0659	300.0872228.0661	0.00-0.88	MS^2^ [300]: 228 (100)*m/z* 300.09>228.06	–	–++	–	+	FLNL (72 Da)MRM
**DP20**	29.27	C_16_H_14_N_2_O_4_	228.0656	228.0661	-2.19	*m/z* 299.10>228.06	–	––+	–	–	FLNLMRM

FL: Full scan (*m/z* 200–1500); NL: neutral loss scan; MRM: multiple reaction monitoring scan; A: pH=4; N: pH=7; B: pH=10; ^a^1E7-03 was incubated in the buffers with different pH for up to 48 hrs. ^b^1E7-03 was incubated in the mouse plasma up to 24 hrs. ^c^ DP8 was detected in the buffers after 24 hrs incubation.

To identify additional 1E7-03 degradation products, 1E7-03 was subjected to various conditions, including incubation in buffers with pH 4, pH 7 and pH 10 for 48 hrs at 37°C. All available DPs were identified by advanced LC/FT-MS/MS analysis that included FL, NL and MRM scans. A total of 20 DPs were identified (Figure 1A and Table 2; see also Supplementary Figures 2-5). Of these 20 DPs, 15 DPs were identified by FL scan, 11 DPs were detected by NL scan, and 5 DPs were present at trace amounts and could only be detected by MRM scans. The amide bonds C_13_–N_14_ and N_14_–C_15_, the ester bond C_10_–O_11_, and C_1_/C_2_, C_3_ on cyclopentene ring (Figure 1A) were identified as labile sites or “hotspots”.

### 1E7-03 stability in cell culture

**Figure 2:**
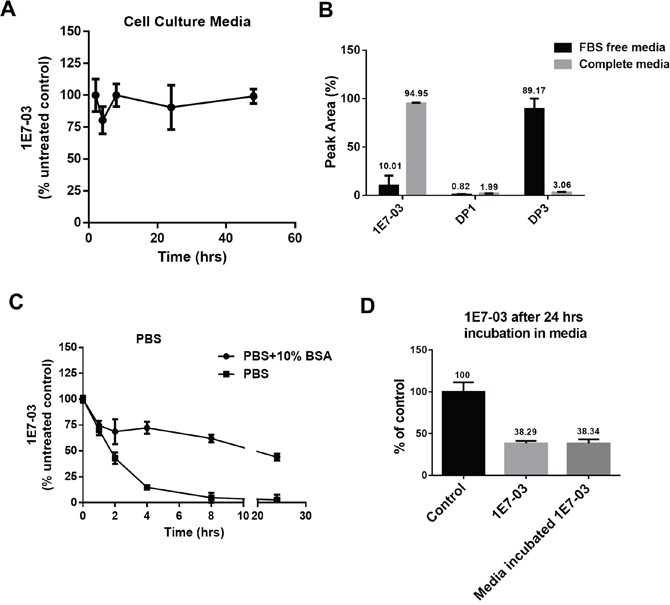
Stability of 1E7-03 in cell culture and the effect of media incubation on its anti-HIV-1 activity **(A)** Stability of 1E7-03 in media supplemented with 10% fetal bovine serum (FBS). 1E7-03 (10 μM) was added to the media and incubated at 37°C for 48 hrs. Samples were collected at different time points, 1E7-03 was extracted and quantified by LC/FT-MS analysis. **(B)** 1E7-03 and DPs degradation in FBS-free media and media supplemented by 10% FBS (complete media). Results at 24 hrs of incubation are shown. **(C)** 1E7-03 degradation in PBS with and without 10% bovine serum albumin (BSA). **(D)** The comparison of untreated 1E7-03 and 1E7-03 incubated in completed media for 24 hrs on single round of HIV-1 replication. CEM T cells were infected with HIV-1-LUC-G and treated with control or media incubated 1E7-03. Percent of luciferase activity is shown. All results were shown by the means ± SD.

In our previous studies, 1E7-03 was used to treat cultured cells infected with HIV-1 [3, 4]. To test the stability of 1E7-03 in cell culture media, the compound was incubated in the complete media for 48 hrs at 37°C and aliquots were collected at different time points. During the incubation, 1E7-03 remained stable and did not undergo degradation (Figure 2A). In contrast, 1E7-03 incubated in serum free media underwent degradation with only 7% of the compound remaining after 24 hrs of incubation at 37°C (Figure 2B). The major degradation product in serum free media was DP3 (91.98%, Figure 2B). As 1E7-03 remained largely intact in complete media (98.6% remaining, Figure 2B), serum albumin may have a protective effect against 1E7-03 degradation. To test this possibility, 1E7-03 was incubated in phosphate buffered saline (PBS) with and without the addition of 10% bovine serum albumin (Figure 2C). 1E7-03 underwent quick degradation in PBS with over 80% of the compound degraded after 4 hrs of incubation (Figure 2C). An addition of 10% BSA stabilized 1E7-03 with 75% of the compound remaining intact after 4 hrs of incubation and over 50% remaining after 24 hrs of incubation (Figure 2C). Thus albumin present in culture media might prevent 1E7-03 from being degraded. We also tested the activity of 1E7-03 after incubation in complete media in a single round HIV-1 infection assay in CEM T cells infected with VSVg-pseudotyped HIV-1 virus expressing luciferase (HIV-1-LUC-G) (Figure 2D). The untreated 1E7-03 and the compound incubated in complete media for 24 hrs inhibited HIV-1 to the same extent (Figure 2D) suggesting that 1E7-03 continued to be active after incubation in complete media.

### Binding of 1E7-03, DP1 and DP3 to PP1 *in vitro*

**Figure 3:**
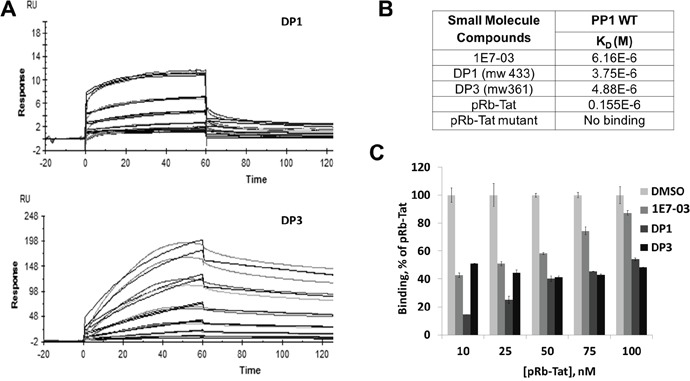
PP1 binding of 1E7-03, DP1 and DP3 Binding of 1E7-03, DP1 and DP3 to recombinant PP1 was measured by surface plasmon resonance. **(A)** Raw data showing binding of DP1 and DP3 to PP1 protein. X axis represents time in seconds and Y axis represents changes in total mass on microchip surface, which was expressed as resonance units. A positive deflection indicated binding of DP1 and DP3 to PP1 immobilized on a microchip surface. Each line represents a different concentration of DP1 and DP3 (0-40 μM). Each concentration was run two times. **(B)** The equilibrium dissociation constants (K_D_) calculated based on a 1:1 binding model. **(C)** Binding competition assay. 1E7-03, DP1 and DP3 compete with pRb-Tat peptide for binding to PP1. Different concentrations of pRb-Tat hybrid peptide were premixed with 1 μM of compounds.

To determine whether the major degradation products of 1E7-03 retained the ability to bind PP1, we analyzed their binding of PP1 *in vitro*. Recombinant PP1 protein was purified as previously described [3] (see purification details in Materials and Methods) and used to analyze the binding of 1E7-03 and chemically synthesized DP1 and DP3 with surface plasmon resonance technology on a Biacore T-200 instrument (Figure 3A). The PP1 was immobilized on a sensor chip CM5 and different concentrations of 1E7-03, DP1 and DP3 were injected over the surface of the chip. Direct binding of the compounds to PP1 was measured in real time and binding affinities were calculated based on a 1:1 binding model. 1E7-03, DP1 and DP3 bound to PP1 with similar low micromolar K_D_ values (Figure 3B). A control PP1 binding peptide containing a retinoblastoma protein-derived phosphopeptide linked to an RVxF-containing sequence derived from HIV-1 Tat [3] (pRb–Tat) showed a 20-40 times higher binding ability (K_D_=0.15×10^-6^, Figure 3B). A mutant pRb-Tat peptide with QACA mutation in the RVxF motif did not bind to PP1 in accord with our previous report [4] (Figure 3B). This result shows that the DPs retained the ability to interact with PP1 *in vitro*.

To determine whether the compounds bound to the RVxF-binding site, a competition assay was performed with the compounds and pRb-Tat peptide previously shown to occupy RVxF-binding on PP1 (Figure 3C). Increasing concentrations of pRb-Tat peptide (10 nM, 25 nM, 50 nM, 75 nM and 100 nM) were added to the compounds that were kept at a constant concentration (1 μM) (Figure 3C). DMSO was used as a vehicle control. All three compounds competed with the pRb-Tat protein for binding to PP1 with DP1 being the best competitor as compared to 1E7-03 or DP3. Thus, Biacore experiments demonstrated a direct binding of 1E7-03 degradation products, DP1 and DP3, to PP1 and a competition with the PP1-binding peptide containing an RVxF motif. DP1 showed a slightly better binding to PP1 *in vitro* and superior competition capability when used at low concentration in comparison to 1E7-03 or DP3.

### Anti-HIV-1 activity of 1E7-03 degradation products

**Figure 4:**
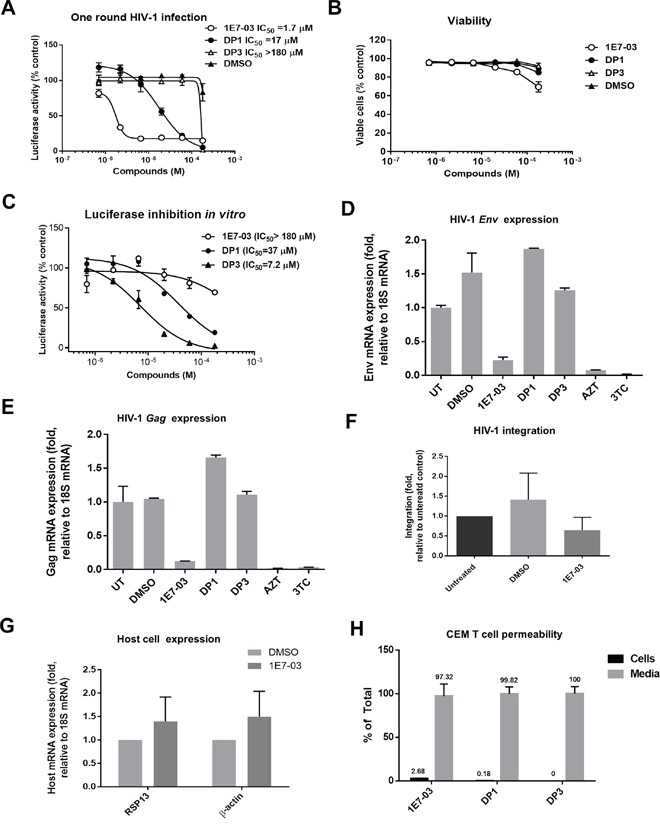
HIV-1 inhibition and cell permeability of 1E7-03, DP1 and DP3 **(A)** Inhibition of HIV-1 in single round of HIV-1 replication. CEM T cells were infected with HIV-1-LUC-G for 24 hrs and then treated for 24 hrs at 37°C with DMSO, 1E7-03, DP1 or DP3. Luciferase activity was then measured.. Percent of luciferase activity is shown. **(B)** Toxicity of 1E7-03, DP1 and DP3 in CEM T cells. CEM T cells were treated with the indicated concentrations of the compounds for 24 h at 37°C and cell viability was determined using trypan blue-based assay. **(C)** The effect of 1E7-03, DP1 and DP3 on luciferase activity. Purified luciferase was incubated with serial dilutions of the compounds or vehicle (DMSO) at room temperature for 10 min. **(D-E)** Inhibition of HIV-1 *env* mRNA (D) and HIV-1 *gag* mRNA (E) expression. RNA was extracted from CEM T cells that were infected with HIV-1-LUC-G for 24 hrs and then treated with DMSO, 10 μM 1E7-03, 10 μM DP1, 10 μM DP3, 5 μM AZT or 5 μM 3TC. RNA was reverse transcribed and *env* and *gag* mRNA was quantified by real-time PCR using 18S mRNA for normalization. N=3 for all panels. The means ± SD are shown. **(F)** The effect of 1E7-03 on HIV-1 integration. CEM T cells were infected with HIV-1-LUC-G and simultaneously treated with 10 μM 1E7-03 or DMSO. DNA was extracted 6 hrs post infection and analyzed by real-time PCR using Alu and Gag primers for HIV-1 DNA detection and β-globin gene as a reference. **(G)** The effect of 1E7-03 on cellular transcription. CEM T cells were treated with 10 μM 1E7-03 and expression of RSP13 and β-actin was quantified by real-time RT-PCR using 18S mRNA for normalization. **(H)** Cell permeability of 1E7-03, DP1 and DP3. Compounds were added to the CEM T cells and incubated for 24 hrs. Amount in the media and intracellular accumulation of compounds was quantified by LC/FT-MS.

To analyze whether DP1 and DP3 retained the ability to inhibit HIV-1 *in vitro*, we analyzed HIV-1 inhibition in CEM T cells infected with HIV-1-LUC-G. While 1E7-03 effectively inhibited HIV-1 (IC_50_ = 1.7 μM), DP1 was 10 times less effective (IC_50_ = 17 μM) and DP3 was not inhibitory (IC_50_ > 180 μM) (Figure 4A). DP1 and DP3 had no effect on cell viability, whereas 1E7-03 showed about 30% viability reduction at the highest tested 180 μM concentration (Figure 4B).

Next, we analyzed the effect of 1E7-03, DP1 and DP3 on purified luciferase activity *in vitro*. While 1E7-03 had no effect on luciferase activity at concentrations below 100 μM, both DP1 and DP3 showed potent luciferase inhibition at IC_50_=37 μM and IC_50_= 7.2 μM, correspondingly (Figure 4C). Thus, the effect of DP1 and DP3 on HIV-1-LUC-G could be due to the inhibition of luciferase and not due to the viral inhibition.

To further analyze whether DP1 and DP3 can inhibit HIV-1, we measured HIV-1 mRNA expression in CEM T cells infected with HIV-1-LUC-G. The expression of HIV-1 *env* mRNA (Figure 4D) and *gag* mRNA (Figure 4E) was significantly reduced in the cell treated with 10 μM 1E7-03. In contrast HIV-1 *env* and *gag* mRNA expression was not affected when DP1 and DP3 were used at 10 μM concentrations (Figure 4D and 4E). DMSO treatment slightly induced *env* expression in accord with previous observations [14, 15]. We observed strong inhibition of *env* and *gag* expression when 5 μM azidothymidine (AZT) or 5 μM lamivudine (3TC) were used (Figure 4D and 4C).

We next tested the effect of 1E7-03 on HIV-1 integration which did not show any significant effect (Figure 4F, *p*=0.97 for DMSO versus 1E7-03). To exclude the “off target” effect of 1E7-03, we also tested the effect of 1E7-03 on expression of cellular genes. We observed slight induction of RSP13 mRNA and β-actin mRNA (Figure 4G), suggesting that 1E7-03 did not inhibit cellular transcription.

Finally, we analyzed whether the reduced HIV-1 inhibitory activities of DP1 and DP3 were due to their inefficient cellular permeability. CEM T cells were incubated with 10 μM 1E7-03, DP1 or DP3 for 24 hrs at 37°C. The compounds remaining in media and taken up by the cells were quantified by LC/FT-MS in media aliquots and cell lysates. 1E7-03, DP1 and DP3 were all detected in media (Figure 4H). While 1E7-03 showed good cell permeability with 3% accumulation in cellular lysates (Figure 4H), DP1 accumulation in the cell lysate was less than 0.2% (Figure 4H) and DP3 was not detected in lysates (Figure 4H). Thus cells accumulated DP1 more than 10 times less efficiently than 1E7-03 which may explain its lower activity against HIV-1. The lack of cellular penetration by DP3 also explains its inability to inhibit HIV-1.

### Effect of 1E7-03 on cellular proteome

**Figure 5:**
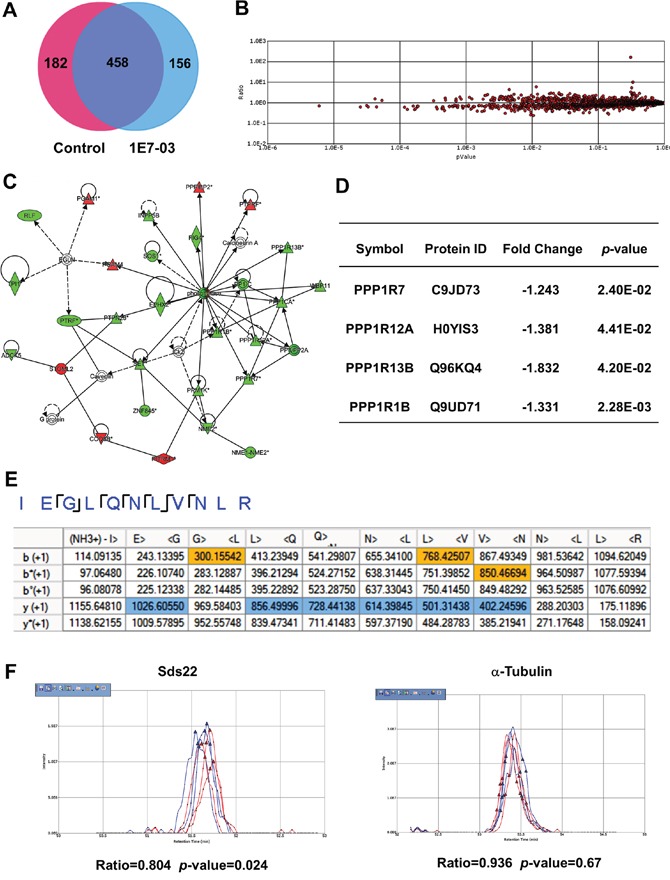
Effect of 1E7-03 on PP1 and its regulatory subunits **(A)** Venn diagram of proteins from CEM T cells treated with 10 μM 1E7-03 or DMSO as a control. Cellular proteins were extracted, reduced, alkylated and trypsinized. Tryptic peptides were analyzed by high resolution mass spectrometry. Proteins were identified by Proteome Discoverer 1.4. **(B)** Quantitative analysis by SIEVE 2.1. The volcano plot shows ratios and corresponding *p*-values of peptides present in 1E7-03 versus DMSO treated samples. **(C)** Quantified proteins were uploaded to the Ingenuity Pathway Analysis software for a Core analysis. A network of PP1 and its regulatory subunits is shown. Up-regulated genes are colored in red and down regulated genes are colored in green. **(D)** Effect of 1E7-03 on the expression of PP1 regulatory submits. **(E)** Consensus view of representative peptides derived from Sds22. Matched b^+1^ and y^+1^ ions are respectively colored by yellow and blue. **(F)** Quantitative analysis of Sds22 expression using SIEVE 2.1. Ion elution profiles are shown in blue for control samples and in red for 1E7-03 treated CEM T cells. Triangles indicate the time points at which MS/MS was conducted. The left panel shows the elution of the Sds22-derived ion. The right panel shows the elution of the α-tubulin-derived ion. The ratios and *p*-values of the ion peaks in control versus 1E7-03 treated groups are shown.

We recently showed that PP1-activating small molecule SMAPP1 induced expression of PP1-regulatory Sds22 subunit in CEM T cells [16]. To determine if 1E7-03 has a global effect on protein expression and specifically on PP1 and its regulatory subunits, we analyzed the proteins expressed in 1E7-03-treated CEM T cells using nano liquid chromatography followed by FT-MS/MS tandem mass spectrometry [16]. We performed a short-gun label free quantitative proteomics analysis on whole cell extracts of 1E7-03-treated CEM T cells. A total of 3606 peptides derived from 796 proteins were identified by Proteome Discoverer 1.4 (Figure 5A). To determine whether the levels of PP1-related proteins were affected by 1E7-03 treatment, we quantified protein expression using a label-free approach. For this purpose, we used SIEVE 2.1 software which allows for the extraction of selected ions and their quantification by integrating their ion elution profiles (Figure 5B). Over 140 proteins were affected by 1E7-03. We used Ingenuity Pathway Analysis (IPA) software to identify a network of PP1-related proteins (Figure 5C). Four PP1 regulatory subunits were downregulated by 1E7-03, including Sds22 subunit encoded by PPP1R7 gene (Figure 5D). Sds22 was identified by the MS/MS analysis of its representative peptides (Figure 5E), and its quantification by SIEVE showed down regulation (Ratio_1E7-03/Control_ =0.804, *p*=0.024, Figure 5F) compared to α-tubulin (Ratio_1E7-03/Control_ =0.936, *p*=0.67, Figure 5F). Thus, PP1-related proteins might be affected by 1E7-03 treatment and contribute to the HIV-1 resistance.

### Antiviral efficacy of 1E7-03 in NSG mice with established HIV-1 infection

**Figure 6:**
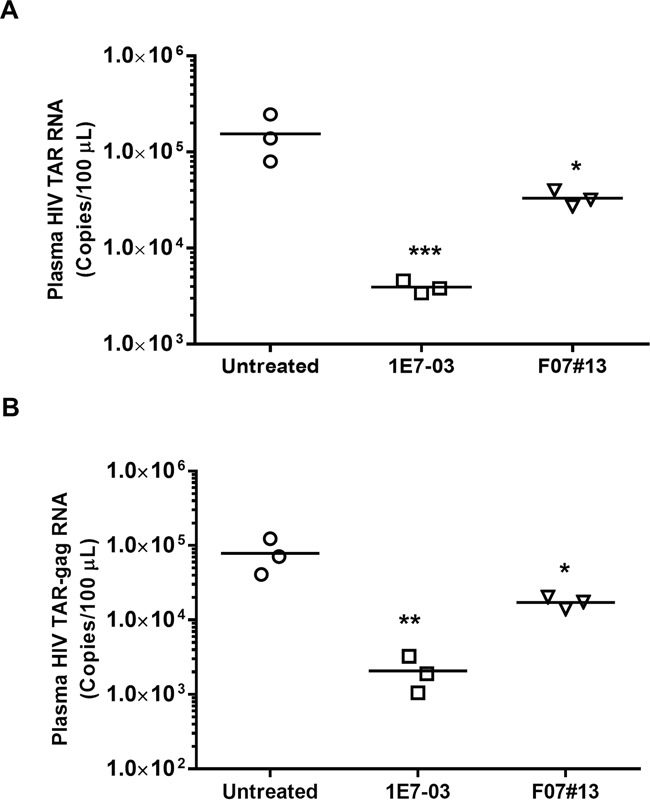
Antiviral efficacy of 1E7-03 in HIV-1 89. 6-infected NSG mice Mice were treated with 3 mg/kg of 1E7-03 or 1.5 mg/kg F07#13 by a single dose intraperitoneal (i.p.) injection After 48 hrs, mice were sacrificed; plasma samples were collected and tested for levels of HIV-1 TAR RNA **(A)** and HIV-1 TAR-gag RNA **(B)**. For quantitative analysis of HIV-1 RNA, total RNA was isolated from blood specimens, treated with RNase-free DNase I and reverse transcribed. Real-time PCR reactions were carried out in triplicates. Each data point represents the blood from a single animal. For all figures, *p< 0.05, **p< 0.01, ***p< 0.001, using unpaired *t* test.

Next, we determined the effect of 1E7-03 on HIV-1 infection *in vivo* using NSG mice infected with the dual tropic HIV-1 89.6. Groups of 3 mice were treated with a single i.p. of 1E7-03 (3 mg/kg) or F07#13 (1.5 mg/kg) at a time point when HIV-1 89.6 replication normally peaks in these animals [13]. 1E7-03 reduced HIV TAR RNA by >40-fold, *p*=0.0001 (from a mean of 10^5.19^ HIV RNA copies per 100 μL of blood in untreated mice to 10^3.59^ copies in 1E7-03 treated mice, Figure 6A) and TAR-*gag* RNA by >39-fold, *p*=0.0014 (from a mean of 10^4.90^ HIV TAR-*gag* RNA copies per 100 μL of blood in untreated mice to 10^3.31^ copies in 1E7-03 treated mice, Figure 6B). In comparison, F07#13 (a Tat mimetic inhibitor) at 1.5 mg/kg only reduced TAR RNA by 4.7-fold, *p*=0.0117 (from a mean of 10^5.19^ HIV TAR RNA copies per 100 μL of blood in untreated mice to 10^4.52^ copies in F07#13 treated mice, Figure 6A) and TAR-gag RNA by 4.6-fold, *p*=0.0110 (from a mean of 10^4.90^ HIV TAR-gag RNA copies per 100 μL of blood in untreated mice to 10^4.24^ copies in F07#13 treated mice, Figure 6B). These findings showed that despite its instability, 1E7-03 retained its anti-HIV-1 inhibitory activity *in vivo* which was comparable to and even exceeded the previously tested F07#13 inhibitor.

### DP1-07, a DP1 analog with improved cell permeability and PK properties

**Figure 7:**
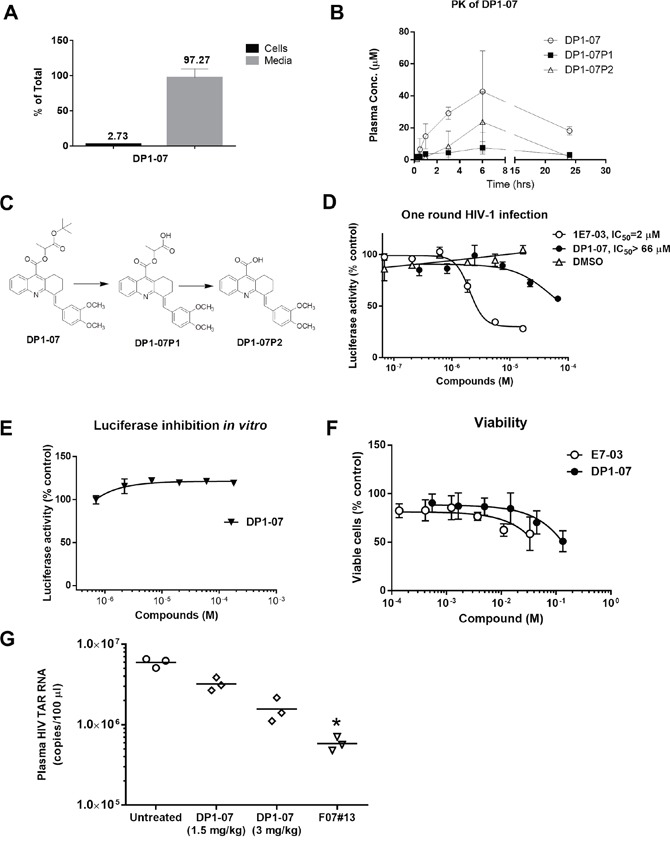
Cellular permeability, pharmacokinetic, and HIV-1 inhibition of the DP1 analog (DP1-07) **(A)** Amount in the media and intracellular accumulation of DP1-07 after 24 hrs of treatment of CEM T cells. Analysis was performed by LC/FT-MS. **(B)** Pharmacokinetic of DP1-07 in mice. Mice were injected i.p. either with 100 mg/kg of DP1-07. The concentrations of DP1-07 and its major DPs in the plasma were quantified by LC/FT-MS. Three mice were used for each time point. The means ± SD are shown. **(C)** Scheme of DP1-07 degradation to two products DP1-07P1 and DP1-07P2. **(D)** HIV-1 inhibition of DP1-07 in single round of HIV-1 replication. CEM T cells were infected with HIV-1-LUC-G. Percent of luciferase activity is shown. **(E)** The effect of DP1-07 on luciferase activity. Purified luciferase was incubated with serial dilutions of DP1-07 or vehicle (DMSO) under room temperature for 10 min. **(F)** Toxicity of DP1-07 in CEM T cells. Cells were treated with calcein-AM for 30 min and calcein fluorescence was measured at 485 nm excitation and 515 nm emission. **(G)** Antiviral efficacy of DP1-07 in HIV-1 89.6-infected NSG mice. Mice were treated with 3 mg/kg and 1.5 mg/kg of DP1-07 by a single dose i.p. injection. After 48 hrs, mice were sacrificed and plasma samples were tested for levels of HIV-1 TAR RNA. Each data point represents the blood from a single animal. *p< 0.05 using unpaired t test.

To improve cellular permeability of DP1, we synthesized DP1-07 compound (see details in Materials and Methods; Supplementary Figure 1) and tested its cellular permeability. CEM T cells were incubated with 10 μM DP1-07 for 24 hrs at 37°C. The amount of DP1-07 in media and its cellular uptake was quantified by LC-MS as described above. DP1-07 showed good cell permeability with 2.7% accumulation in the cellular lysate (Figure 7A), which was similar to the permeability of 1E7-03 compound. Next, we carried out PK of DP1-07 in the mice. The time-dependent plasma concentrations of DP1-07 and its pharmacokinetic parameters were shown in Figure 7B and Table 1. As expected, DP1-07 showed good stability and pharmacokinetic properties (Figure 7B). DP1-07 (18 μM) was present after 24 hrs post-injection in the collected murine blood (Figure 7B). Of note, we were able to inject a higher amount of DP1-07 because of its better solubility. DP1-07 reached the peak plasma concentration in mice at approximately 6 hrs (*T*_max_) with a concentration (*C*_max_) of 42.71 μM. The area under the plasma concentration-time curve (*AUC*_last_) was found to be 705.61 μM·hr (Table 1). Using LC/FT-MS analysis, we observed two major products of DP1-07 degradation, DP1-07P1 and DP1-07P2 (Figure 7B-7C). The plasma concentration of DP1-07P1 was low at each time points analyzed. DP1-07P2 accumulation paralleled DP1-07 kinetics pattern but its concentration was much lower than DP1-07 (*C*_max_ =23.71 μM). Thus, DP1-07 compound exhibited improved cell permeability and PK properties over 1E7-03.

### HIV-1 inhibition by DP1-07 *in vitro* and *in vivo*

To test the biological activity of DP1-07, we analyzed its effect on HIV-1 replication *in vitro* and *in vivo*. DP1-07 was not efficient in the inhibition of single round HIV-1 replication in CEM T-cells infected with HIV-1-LUC-G (Figure 7D, IC_50_ >66 μM). It did not inhibit luciferase *in vitro* (Figure 7E). DP1-07 showed reduced toxicity in CEM T cells compared to 1E7-03 (Figure 7F). To determine if DP1-07 inhibits HIV-1 *in vivo*, single doses of DP1-07 at 3 mg/kg and 1.5 mg/kg were given to HIV-1_89.6_ infected NSG mice by i.p. The F07#13 (1.5 mg/kg) was used as a positive control. DP1-07 reduced HIV TAR RNA from a mean of 10^6.8^ copies to 10^6.2^ copies at 3 mg/kg (3.8-fold, *p*=0.6617, Figure 7G) and to 10^6.5^ copies at 1.5 mg/kg (1.8-fold, *p*=0.7477, Figure 7G), while F07#13 reduced HIV TAR RNA by 10.2-fold, *p*=0.0422 (Figure 7G). Thus, DP-07 was not an efficient HIV-1 inhibitor *in vitro* and showed reduced efficiency *in vivo* compared to F07#13 or 1E7-03, despite its improved cellular permeability and PK.

### Stability profiles of 1E7-03 in plasma and liver microsomes from various species

**Figure 8:**
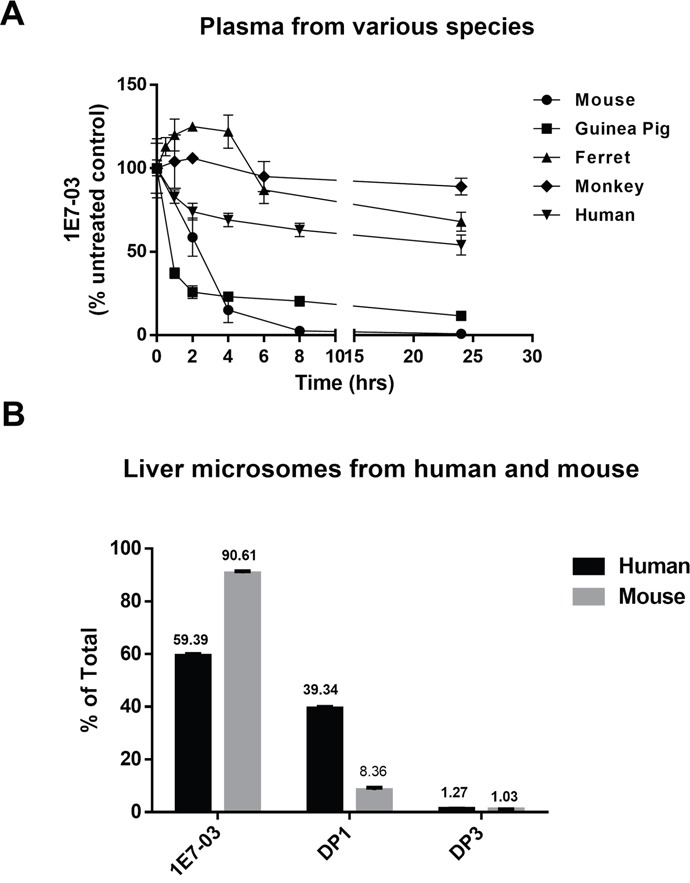
Stability of 1E7-03 in plasma and liver microsomes from different species **(A)** Stability of 1E7-03 in plasma from mouse, guinea pig, ferret, monkey and human. 1E7-03 dissolved in DMSO (10 mM stock solution) was mixed with plasma to the final concentration of 10 μM and incubated at 37°C. Samples were collected at different time points for up to 24 hrs and analyzed by LC/FT-MS. **(B)** Stability of 1E7-03 in liver microsomes from mouse and human. 1E7-03 dissolved in DMSO (10 mM stock solution) was mixed with liver microsomes to the final concentration of 10 μM and incubated at 37°C for 1 hr. Samples were processed and analyzed by LC/FT-MS. All experiments were run parallel in triplicates.

Hydrolysis of compounds containing amides and esters by plasma enzymes [17] may vary in different species [18]. Thus we investigated the stability of 1E7-03 in plasma obtained from various species, including guinea pig, ferret, monkey and human (Figure 8A). For comparison, the corresponding plasma concentration-time profile of 1E7-03 in mouse plasma was also shown (Figure 8A). 1E7-03 underwent quick degradation in guinea pig plasma similar to the mouse plasma with about 80% of the compound being degraded after 4 hrs of incubation. In contrast, 1E7-03 remained relatively stable in human, monkey and ferret plasma with over 50% remaining even after 24 hrs of incubation (Figure 8A). Liver microsomes represent a critical experimental model to estimate the drug metabolic fates *in vivo* [19]. We tested the stability of 1E7-03 in liver microsomes obtained from humans and mice. After 1 hr of incubation, 1E7-03 remained stable in mouse liver microsomes (91% remaining, Figure 8B), while 39% of 1E7-03 in human liver microsomes was degraded and converted into DP1 (Figure 8B).

## DISCUSSION

Prior to the first administration of a drug in humans, it is typically tested in small laboratory animals to determine its *in vivo* activity, drug pharmacokinetics and metabolic stability, all of which are critical in the selection of drug candidates [20]. Comprehensive analysis of *in vivo* activity and metabolic behavior can provide effective guidance for the further structure optimization of lead compounds and subsequent preclinical or clinical studies.

We first determined 1E7-03 stability both *in vivo* and *in vitro*. 1E7-03 rapidly degraded into its main metabolite DP1 with a *t*_1/2_ of 3.39 hrs when it was given to mice by a single dose i.p. Accompanied by the degradation of 1E7-03, DP1 level increased reaching *C*_max_ of 9.09 μM after 3 hrs of injection. This observation suggested that DP1 could potentially contribute to the *in vivo* anti-HIV activity of 1E7-03. The dynamic stability plot of 1E7-03 in mouse plasma showed a very similar profile with its PK profile. Thus hydrolysis in plasma is likely to be the major route for clearance of 1E7-03. Rapid DP1 formation indicates that the amide bond C_13_–N_14_ in 1E7-03 is a hotspot that is being cleaved in murine plasma. In contrast, 1E7-03 showed good stability in complete but not the serum-free cell culture media, which could be partly attributed to the presence of albumin in the media. Over 90% 1E7-03 remained intact in cell culture media after 48 hrs of incubation. Moreover, the 1E7-03 incubated for 24 hrs in media and then added to HIV-1 infected cells showed the same level of HIV-1 inhibition as the freshly prepared 1E7-03, indicating that 1E7-03 was directly responsible for the anti-HIV-1 activities observed in the cultured cells. While the stability of some compounds is pH-dependent, we did not detect any effect of pH on 1E7-03 degradation pattern tested in buffers with pH 4–10 (data not shown).

To understand whether DPs retained the ability to target PP1 and inhibit HIV-1, we analyzed their PP1-binding affinities and their biological activities. Both DP1 and DP3 bound efficiently to PP1 *in vitro* (K_D_ ~ 4 μM), with a slightly higher affinity than 1E7-03 (K_D_ = 6.2 μM). The affinity of PP1-binding RVxF motif containing peptide pRb-Tat (K_D_ = 0.16 μM) was significantly higher than the small compounds affinity likely because of an extended binding site in addition to the RVxF binding motif. Unfortunately, our numerous attempts to co-crystallize PP1 with 1E7-03, DP1 or pRb-Tat were not successful (Wolfgang Peti and Sergei Nekhai, unpublished observations). Thus another approach was needed to elucidate the interaction of 1E7-03 and its DPs with PP1. We performed a competition assay of 1E7-03, DP1 and DP3 with pRb-Tat measured by surface plasmon resonance which showed DP1 being the best *in vitro* competitor. It reduced the binding of pRb-Tat to PP1 by 10-fold compared to only about a 2-2.5 -fold reduction by 1E7-03 and DP3. Thus, a future compound based on the DP1- structure may have a strong potential as a PP1-targeting molecule. Unfortunately, DP1 was not efficient as a HIV-1 inhibitor. Moreover, its inhibition of HIV-1-LUC-G could be partly attributed by its inhibition of luciferase. This could explain why DP1 did not inhibit expression of HIV-1 *env* and *gag* mRNA at a 10 μM concentration. The apparent reason for the reduced activity of DP1 was its 10-fold reduced cellular permeability compared to 1E7-03. Therefore, the reduced intracellular concentration of DP1 may explain its reduced antiviral activity.

Our previous studies showed that HIV-1 Tat interacts with PP1 and translocates it to the nucleus [7]. The PP1-targeting HIV-1 inhibitor 1E7-03 binds to PP1 and disrupts the Tat-mediated translocation of PP1 into the nucleus in cultured cells. By targeting PP1, we also developed SMAPP1, an activator of the latent HIV-1 provirus [16]. CDK9 Ser90 has been reported to be phosphorylated by CDK2, which induces HIV-1 transcription [21, 22]. We showed SMAPP1 could increase Ser90 phosphorylation of CDK9 by reducing the dephosphorylation activity of PP1 or indirectly activating CDK2 to induce HIV-1 transcription. The protein expression analysis in T cells showed that SMAPP1 upregulates the PP1-regulatory subunit, Sds22, which is an evolutionarily conserved ancient interactor of PP1 that along with Inhibitor-3 forms a complex with PP1 and helps translocate it to the nucleus [16]. In the current study, label free quantitative proteomics analysis of 1E7-03 in CEM T cells showed that the expression of Sds22 is downregulated by 1E7-03 treatment during HIV-1 infection, suggesting that expression of Sds22 may change the cellular distribution of PP1 and potentially deregulate translocation of PP1 between cytoplasm and nucleus thus impacting PP1 availability for Tat recruitment and CDK9 dephosphorylation.

NSG mouse model is a useful platform for the preclinical evaluation of antiviral efficacy of HIV-1 targeting therapeutics. This mouse model allows for the establishment of systemic HIV infection and plasma viremia after the initial HIV infection by multiple routes [23]. The successful HIV-1 inhibition by 1E7-03 in humanized mice clearly demonstrates the antiviral potency of this novel PP1-targeting small molecule. A single dose of 1E7-03 injected i.p. at 3 mg/kg reduced plasma HIV TAR RNA and TAR-gag RNA over 40-fold and 39-fold, respectively. These inhibitory effects exceed the effect of F07#13 (about 5-fold reduction at 1.5 mg/kg dose), an HIV-1 transcription inhibitor that targets an interface between a CDK and cyclin needed for Tat activated transcription [13]. F07#13 also suppresses HIV-1 by about 7-fold in the humanized Rag2^-/-^γc^-/-^ mice at 4 mg/kg [13].

To improve cellular permeability of DP1, we synthesized its analog, DP1-07. This analog demonstrated a cellular permeability similar to 1E7-03, and improved PK properties compared to 1E7-03. Despite that, DP1-07 did not show efficient HIV-1 inhibition (IC_50_>66 μM) in the cellular assay. *in vivo*, DP1-07 only reduced HIV TAR RNA by 3.8-fold at 3 mg/kg in humanized mice, which was about 10-times less efficient than 1E7-03. The lack of improvement of HIV-1 inhibition by DP1-07 over DP1 may be due to the inability of DP1-07 to penetrate to the nucleus or the requirement of the full side chain of 1E7-03 for its anti-HIV activity.

Compounds with certain functional groups including amides, esters, lactams, sulfonamides, tend to undergo hydrolysis by plasma or liver enzymes, exhibiting rapid clearance, short half-lives and consequently poor *in vivo* activity [17, 19]. Thus, we tested stability of 1E7-03 in plasma obtained from different species, including human, monkey, ferret and guinea pig. 1E7-03 was stable in human, monkey and ferret plasma and unstable in guinea pig plasma. This could be due to high expression levels of esterases and amidases in rodents [18]. We also tested the stability of 1E7-03 in liver microsomes from human and mouse. 1E7-03 was less stable in liver microsomes from humans than mice.

In summary, 1E7-03 represents the first example of a PP1-targeting small molecule that significantly inhibits HIV-1 in humanized mice. Taking into account the metabolic stability of 1E7-03 in plasma and liver microsomes, further structural modification or optimized drug delivery to improve stability in the liver is pivotal for future testing in non-human primate animal models.

## MATERIALS AND METHODS

### Chemicals and reagents

1E7-03 (purity above 98%) was synthesized by Enamine (Kiev, Ukraine) as previously described [3]. Acetonitrile and water containing 0.1% formic acid (FA) were Optima LC/MS grade (Fisher Scientific, Fair Lawn, NJ). High-purity nitrogen (99.9%) was purchased from Roberts Oxygen Co, Inc. (Rockville, MD). Other reagents were of analytical grade. Dimethyl sulphoxide (DMSO), acetone, hydrochloric acid and sodium hydroxide were from Fisher Scientific (Fair Lawn, NJ). Phosphate buffered saline (pH 7.4) was from Life Technologies (Grand Island, NY). Sodium acetate (pH 5.2) was from Quality Biological (Gaithersburg, MD).

### Cells and media

CD4+ T cells (CEM) were purchased from the American Type Culture Collection (Manassas, VA). The CEM cells were cultured in RPMI medium (Invitrogen) containing 10% FBS and 1% antibiotic solution (penicillin and streptomycin).

### Pharmacokinetic analysis in mice

The animal facilities and protocols were approved by Howard University Institutional Animal Care and Use Committee (IACUC). All experimental procedures were in accordance with the NIH Guidelines for the Care and Use of Laboratory Animals. BALB/cJ male mice (8 weeks old, 25–30 g) were purchased from Jackson Laboratory (Bar Harbor, ME, USA) and housed under pathogen-free conditions in HEPA-filtered cages and kept on a 12 h light/dark cycle. Compounds were administered to mice via intraperitoneal (i.p.) injection with maximum achievable doses of 30 mg/kg 1E7-03 (100 μl injection of 8 mM 1E7-03 in 80% DMSO/20% saline) and 100 mg/kg DP1-07 (100 μl injection of 100 mM DP1-07 in 40% propylene glycol/10% DMSO/50% saline). Blood samples were collected at different time points (15 min, 30 min, 1 hr, 3 hr, 6 hrs and 24 hrs) for each group (n=3 mice per group). For each sample, 20 μl of plasma was mixed with 80 μl cold acetone, and centrifuged at 13,000 rpm for 5 min. The supernatant was transferred to a clean test tube and dried in a SpeedVac concentrator. The residue was re-constituted in 50 μl of acetonitrile for LC-MS analysis. Calibration curves were prepared by spiking 1E7-03, DP1, DP3 and DP1-07 into the drug-free mouse plasma and subjected to similar sample extraction procedures. The plasma concentration–time profile of each analyte was constructed and pharmacokinetic parameters were determined by non-compartmental method using Phoenix WinNonlin Version 5.3 (Pharsight Corporation, Mountain View, CA).

### Generation of NSG-BLT mice and treatment of 1E7-03 and DP1-07

NOD.Cg-Prkdcscid Il2rgtm1Wjl/SzJ (NSG) mice initially purchased from Jackson Laboratories were bred and maintained in the Animal Core Facility at George Mason University (Manassas, VA). NSG mice were generated by i.p. injection of cryopreserved human CD34+ hematopoietic stem progenitor cells (HSPC) (500,000 cells per mouse) isolated from the autologous fetal liver [23]. Anesthetized mice were inoculated by direct injection 16 weeks after implantation with 50 μl of HIV-1 89.6 (1,000 TCID50) or RPMI 1640 medium (mock infection). Upon reconstitution and presence of virus in the plasma (typically 4-8 weeks), the animals were treated with a single i.p. of 1E7-03 (3 mg/kg), DP1-07 (1.5 mg/kg or 3 mg/kg) or F07#13 (1.5 mg/kg) [13]. Blood was collected by tail vein after 48 hrs and transferred into microcentrifuge tubes containing 330 mM EDTA. Each data point represents the blood from a single animal. Plasma was removed after low-speed centrifugation for 5 min at room temperature and stored at -80°C. Viral RNA was extracted from plasma using the QIAamp Viral RNA Mini Kit (Qiagen). For quantitative analysis of HIV-1 RNA, total RNA was isolated from blood specimens of HIV-1-infected humanized mice. RNA was isolated using Trizol Reagent (Invitrogen, Carlsbad, CA) according to the manufacturer's protocol. A total of 1.0 μg of RNA was treated with 0.25 mg/ml DNase I RNase-free (Roche, Mannheim, Germany) for 60 min in the presence of 5 mM MgCl_2_, followed by heat inactivation at 65 °C for 15 min. A 200–250 ng aliquot of total RNA was used to generate cDNA with the GoScript Reverse Transcription System (Promega, Madison, WI) using TAR-specific reverse primer TAR+59R: 5′- CAACAGACGGGCACACACTAC -3′. Subsequent quantitative real-time PCR analysis was performed with 2 μl of undiluted RT reaction mixes. The iQ Supermix (Bio-Rad, Hercules, CA) was used with the primers specific for (1) HIV-1 TAR: TARfll-F: 5′-GGTCTCTCTGGTTAGACCAGATCTG-3′ and TAR+59R as above; (2) HIV-1 TAR-*gag*: Gag 1625R: 5’- GCTGGTAGGGCTATACATTCTTAC -3’ and TAR+59R as above. Real-time PCR reactions were carried out in triplicate.

### Synthesis of DP1, DP3 and DP1-07

Synthesis of DP3 began with a Pfitzinger reaction between isatin (1) and cyclopentanone resulting in the formation of tricyclic acid 2 (see schematics in Supplementary Figure 1A). Reaction of the latter with veratraldehyde furnished the desired product, DP3. Chemical synthesis of DP1 is described in Supplementary Figure 1B. It began with a base-mediated coupling of DP3 with secondary bromide 4 resulting in the formation of diester 5. Chemoselective deprotection of the *t*-butyl ester resulted in the formation of DP1. Synthesis of DP1-07 is described in Supplementary Figure 1C. Briefly, it began with a Pfitzinger reaction between isatin and cyclohexanone resulting in forming of tricyclic acid (intermediate product 6). Reaction of the product 6 with veratraldehyde resulted in formation of intermediate product 7. The intermediate 7 reacted with secondary bromide 4 resulted in the formation of desired product DP1-07.

### LC/FT-MS analysis

A 10 μl aliquots from each sample were loaded to a LC-20AD nano HPLC system (Shimadzu Corporation, Columbia, MD, USA) coupled to LTQ XL Orbitrap mass spectrometer (Thermo Fisher Scientific) with the installed Xcalibur software (version 2.0.7, Thermo Fisher Scientific). Liquid chromatography was carried out on an in-house made nano-HPLC column (Polymicro Technologies Inc., Phoenix, AZ, USA) packed with reverse phase PolySulfoethyl A, 5μM, 200 Å (PolyLC Inc., Columbia, MD, USA). Mobile phase A was 0.1% formic acid in water and mobile phase B - 0.1% formic acid in acetonitrile. The elution was performed at a flow rate of 600 nl/min over 40 min using a multisegment linear gradient of mobile phase B as follows: 0–6.02 min, 1% B; 6.02–6.11 min, 1–2% B; 6.11–20 min, 2−80% B; 20–25 min, 80% B; 25–30 min, 80–85% B; 30–31 min, 80–2% B; 31–40 min, 2% B (v/v). The Orbitrap was operated under data-dependent acquisition mode. The spray voltage, capillary temperature and capillary voltage were set to 2.0 kV, 200 °C, and 39.5 V, respectively. Full-scan mass spectra were acquired in Orbitrap over 150−1500 *m/z* with a resolution of 30 000, followed by MS^n^ scans by CID activation mode. The precursor ions were fragmented with collision energy of 18 with activation Q of 0.25 and an activation time of 30 ms. Dynamic exclusion was enabled with a repeat count of 2, a repeat duration of 15 s, exclusion duration of 20 s, an exclusion list size of 50. The mass spectrometer was also operated in NL scan of *m/z* 18.01, 45.05, 71.03 and 72.02 and MRM mode. The MRM transitions were controlled by a segmented program in order to short the scan cycle and increase sensitivity.

### LC/FT-MS instrument validation

1E7-03, DP1 and DP3 were dissolved in DMSO (10 mM) and then diluted in acetonitrile to prepare stock solutions (30 μM). The 1E7-03, DP1 and DP3 stock solutions were mixed and then serially diluted to produce calibration standard solutions (10, 5, 1, 0.5, 0.1, 0.05, and 0.01 μM for each compound). The analysis was carried out in triplicates. The peak area versus concentration data were analyzed by least squares linear regression analysis. The intra- and inter-day precisions were established by analyzing drug solution at 1 μM concentration, and six individual analyses were conducted on the same day and on three consecutive days. Accuracy was determined by analyzing a known concentration of drug solution spiked with samples from mouse serum, cell culture media and buffer (pH=7.0) incubations in triplicate and then determining the percent recovery. The limit of detection (LOD) and limit of quantification (LOQ) were estimated at a signal-to-noise of 3:1 and 10:1, respectively, by injecting a series of diluted solutions with known concentrations.

### Analysis of 1E7-03 degradation in plasma

1E7-03 dissolved in DMSO (10 mM stock solution) was mixed with mouse plasma (Sigma-Aldrich), guinea pig plasma (Sigma-Aldrich), ferret plasma (BioreclamationIVT), monkey plasma (courtesy of Binhua Ling, PhD, Tulane University) and human plasma (collected from heathy volunteer by the Center for Sickle Cell Disease at Howard University) to the final concentration of 10 μM and incubated at 37°C. Aliquots were collected at different time points for up to 24 hrs. The resulting sample (60 μl) was mixed with 240 μl of cold acetone, vortexed for 2 min, kept at –20°C for 30 min, and then the precipitated protein was removed by centrifugation at 13,000 × g for 5 min. The supernatant was transferred to a clean test tube and evaporated to dryness using a SpeedVac concentrator. The residue reconstituted in 60 μl of acetonitrile for LC/FT-MS analysis. All experiments were run parallel in triplicates.

### Analysis of 1E7-03 degradation in plasma and liver microsomes

The metabolic stability of 1E7-03 was also investigated using a pool of human and mouse liver microsomes (HMS9PL, MSS9PL, Thermo Scientific) following manufacturer's instructions. In brief, incubations were conducted at 37 ± 1°C in mixtures containing 5 μl of human or mouse liver microsomes (20 mg/ml), 183 μl of potassium phosphate buffer (pH 7.4, 100 mM) and 2 μl of 1E7-03 (10 mM sock solution of 1E7-03 in DMSO was diluted into 1 mM by PBS). The mixture was pre-incubated for 5 min, then initiated the reactions with the addition of 10 μl 20 mM NADPH (Sigma Aldrich). The reactions were terminated after 1 hr incubation by adding 800 μl cold acetone. The mixture was kept at 4°C for 30 min, and the precipitated protein was removed by centrifugation (13, 000 g for 10 min at 4°C). A 250 μl aliquot of supernatant was transferred to a clean test tube and evaporated to dryness using a SpeedVac concentrator. The residue was reconstituted in 50 μl of acetonitrile with 0.1% FA for LC/FT-MS analysis.

### Stability of 1E7-03 in cell culture media and PBS

CEM T cells were seeded in 24-well plates (200,000 cells/well). On the next day cells were treated with 10 μM 1E7-03. Media (100 μl) was collected at different time points during 48 hrs and total protein was precipitated by 400 μl of cold acetone and centrifuged at 13,000 × g for 5 min. The supernatant was collected and evaporated to dryness using a SpeedVac concentrator. The dry pellet was reconstituted in 100 μl of acetonitrile for LC/FT-MS analysis. In addition, 1E7-03 (10 μM) was incubated at 37°C in serum-free media and complete media without cell treatment. Media of 100 μl was collected after 24 hrs of incubation. The stabilities of 1E7-03 (10 μM) at phosphate buffered saline (*PBS)* and PBS with the addition of 10% bovine serum albumin were also investigated at 37°C incubation. Samples (100 μl) were collected at different time points up to 24 hrs. All samples were prepared by the same above-mentioned procedure for LC/FT-MS analysis.

### Effect of pH on 1E7-03 degradation

1E7-03 (10 mM) stability at various pH was tested at 37°C in sodium acetate-acetic acid buffer (pH=4), NaH_2_PO_4_/Na_2_HPO_4_ buffer (pH=7) and NaHCO_3_/NaOH buffer (pH=10). Samples (100 ml) were collected at different time points up to 48 hrs, and evaporated to dryness using a SpeedVac concentrator immediately. The pellets resolved in 100 μl of acetonitrile, vortexed for 2 min, then centrifuged at 13,000 × g for 5 min. The supernatant was transferred to a clean tube for LC-MS analysis.

### PP1 expression

PP1 was purified as previously described [3]. BL21 (DE3) Escherichia coli cells (Invitrogen, Carlsbad, CA, USA) were -transformed with a vector expressing human PP1α (residues 7–300) and pGR07, which expresses GroEL/GroED chaperones (both gifts from Dr Mathieu Bollen and Monique Beullens, KU Leuven, Belgium). The cells were grown in media supplemented with 1 mM MnCl2 at 30°C to an A_600_ =0.5. Arabinose (2 g per L) was added to induce expression of the GroEL/GroES chaperones. When A_600_ =1 was reached, the cells were transferred to 10°C and PP1 expression was induced with 0.1 mM Isopropyl β-D-1-thiogalactopyranoside for 20 hrs. Harvested cells were lysed using sonication in a solution containing in 50 mM Tris-HCl (pH 8.0), 5 mM imidazole, 700 mM NaCl, 1 mM MnCl2, 0.1% Triton X-100 (v/v) and protease inhibitors. His-tagged PP1 was purified using a Ni-NTA IMAC column (Qiagen, Valencia, CA, USA). Purified PP1 was dialyzed and stored at −70°C in 50 mM Tris-HCl (pH 8.0), 5 mM imidazole, 700 mM NaCl and 1 mM MnCl_2_. PP1 activity was then assayed as previously described [4].

### Surface plasmon resonance (SPR)

The SPR measurements were conducted on Biacore T200 instrument (GE Healthcare, Piscataway, NJ) at 25°C. Recombinant PP1 was immobilized on a CM5 chip by amine coupling (GE Healthcare). PP1 (200 nM) was captured on flow cell 2 in 10 mM acetate buffer, pH 5.0, supplemented with 2 mM MnCl_2_. The average amount of PP1 immobilized on the surface was 3160 RU. Flow cell 1 was used as a reference surface to subtract background signal. Also injections of the buffer alone were used to provide double reference subtraction. To measure the direct binding of small molecules to PP1, the two flow cells of the sensor chip were primed with running buffer (0.01 M HEPES pH 7.4, 0.15 M NaCl, 0.005% v/v Surfactant P20, 1% DMSO and 2 mM MnCl_2_). For binding and kinetics experiments, all compounds were diluted in the running buffer, at 40 μM, 20 μM, 10 μM, 5 μM, 2.5 μM, 1.25 μM and 0 μM and then passed over the two flow cells at a flow rate of 100 μl/min for 60 sec. The number of response units was recorded after the subtraction of the reference flow cell's value (Fc2-1). Two repetitions were performed for each injection. Data were analyzed using the BiaEvaluation software of Biacore with a 1:1 binding model. For the competition assay, the hybrid peptide consisting of parts of pRb and Tat protein sequences, HIPR(pS)PYKFPSSPLRKKCCFHCQVCFITK (with a single serine amino acid phosphorylated) was used at 10 nM, 25 nM, 50 nM, 75 nM and 100 nM. All the small molecular weight compounds were used at 1 μM.

### Cell viability assays

CEM T cells were cultured in 96-well plates at 37°C and incubated with the indicated compound concentrations for 24 hrs. Cell viability was determined using trypan blue-based assay. The cells were supplemented with 0.2% trypan blue, transferred to a plastic disposable counting chamber and counted on a TC10 Automatic Cell Counter (Bio-Rad).

To assess cytotoxicity with calcein, media was removed and the cells washed with PBS in order to remove serum esterase activity that may cause an increase in fluorescence through the hydrolysis of calcein-AM. Cells were then supplemented with 0.2 μM calcein-AM (Molecular Probes, Invitrogen) for 10 min at 37°C. Fluorescence was measured using the luminescence spectrometer described above implementing 495 nm excitation and 515 nm emission filters.

### Luciferase assays

CEM T cells were infected with VSVG-pseudotyped pNL4-3.Luc.R-E-virus (HIV-1-LUC-G) prepared as previously described [22] and cultured in 96-well white plates ( 1.25 × 10^6^ cells/ml, 100 μl/well) at 37°C and 5% CO_2_. The cells were treated with serial dilutions of the compounds or vehicle (DMSO) and incubated overnight. At 24 hrs post infection, 100 μl of reconstituted luciferase buffer (Luclite Kit, Perkin Elmer) was added to each well, incubated for 10 min and luminescence was measured using Glo-Max Microplate Multimode reader (Promega).

QuantiLum Recombinant Luciferase (20 ng/ml in PBS) was added into 96-well plate (100 μl/well) and incubated with serial dilutions of the compounds or vehicle (DMSO) under room temperature for 10 min. 100 μl of reconstituted luciferase buffer (Luclite Kit, Perkin Elmer) was added to each well, incubated for another 10 min and luminescence was measured using Glo-Max Microplate Multimode reader (Promega).

### Quantitative RT PCR

For quantitative analysis of HIV-1 RNA, CEM T cells were infected with HIV-1-LUC-G virus. Cells were treated with 10 μM 1E7-03, 10 μM DP1, 10 μM DP3, 5 μM AZT or 5 μM 3TC and control cells were untreated or treated with DMSO. Total RNA was isolated using Trizol Reagent (Invitrogen, Carlsbad, CA) according to the manufacturer's protocol. Total RNA (100 ng) was reverse transcribed to cDNA using Superscript™ RT-PCR kit (Invitrogen, Carlsbad, CA). Hexamers and oligo-dT were used as primers. For real-time PCR analysis, cDNA was amplified using Roche LightCycler 480 (Roche Diagnostics) and SYBR Green1 Master mix (Roche Diagnostics). PCR was run for 45 cycles and each cycle included denaturation at 95°C for 10 seconds, annealing at 60°C for 10 seconds, and extension at 72°C for 10 seconds. Quantification of *env* and *gag* was carried using 18S RNA as a normalization standard. Quantification of housekeeping genes, β-actin and RSP13 was carried relative to 18S RNA. Mean Cp values were determined and ΔΔCt method was used to calculate relative expression levels. Unpaired *t*-test was used to test statistical significance.

The following primer sequences were used: *env*, forward - CCTTTGAGCCAATTCCCATACATT, reverse-GACGTTTGGTCCTGTTCCATTGAACGT; *gag*, forward- ATAATCCACCTATCCCAGTAGGAGAAAT, reverse- TITGGTCCITGTCITATGTCCAGAATGC; β-actin, forward- CTCCCAAAGTGCTGGGATTA, reverse-CAAAGGCGAGGCTCTGTG; RSP13, forward- AGATGTGGGAAGGTTGGTGG, reverse- TTTCTCGAGCAGTACCTATCTGG; and 18SrRNA, forward- GCTGTTGCTACATCGACCTTT, reverse- CTCCAGGTTTTGCAACCAGT.

To assay HIV-1 integration, CEM T cells were infected with HIV-1-LUC-G virus and treated with 10 μM 1E7-03 or DMSO. Total DNA was extracted from cells using lysis buffer (10 mM Tris-HCl pH 8.0, 10 mM EDTA, 5 mM NaCl, 200 μg/ml proteinase K). The cells were lysed for 20-30 min at room temperature and proteinase K was inactivated by heating to 95°C for 5 min. For the real-time PCR analysis, 100 ng DNA was amplified using Roche Light Cycler 480 (Roche Diagnostics) and SYBR Green1 Master mix (Roche Diagnostics). PCR was carried with initial preincubation for 5 min at 45°C and then for 3 in at 95°C followed by 45 cycles of denaturation at 95°C for 15 sec, annealing and extension at 60°C for 45 sec, and final extension at 72°C for 10 sec. Integrated HIV-1 DNA was amplified using the following primers: Alu forward 5'-GCCTCAATAAAGCTTGCCTTGA-3', and Gag reverse 5'-GCTCTCGCACCCATCTCTCTCC-3'.

### Cellular permeability assay

CEM T cells were seeded in 6-well plates (0.5 × 10^6^ cells/ml) and treated by 10 μM of the indicated compounds. Two samples were generated for each compound (in triplicates) obtained from cellular lysate and media sample. Cell culture media samples (100 μl) were collected from each well after 24 hrs of incubation and total protein was precipitated by the addition of 400 μl cold acetone. Protein pellets were washed three times with PBS to remove any compound that had not permeated into the cells. To obtain cell lysates, the cells were suspended in 300 μl cold whole cell lysis buffer (50 mM Tris-HCl, pH 7.5, 0.5 M NaCl, 1% NP-40, 0.1% SDS) and sonicated. A 100 μl aliquot of the cell lysate was combined with 400 μl cold acetone to precipitate proteins. All samples were centrifuged at 13,000 × g for 5 min. Supernatants containing compounds and their metabolites were collected and evaporated to dryness on SpeedVac concentrator. The dry pellet was reconstituted in 50 μl acetonitrile for LC/FT-MS analysis.

### Label free quantitative proteomics analysis

CEM T cells were infected with VSVG-pseudotyped pNL4-3.Luc.R-E-virus (HIV-1-LUC-G) and cultured in 100 mm culture plates (1.25 × 10^6^ cells/ml) containing 10 ml of supplemented RPMI media at 37°C and 5% CO_2_. The cells were treated with 10 μM of 1E7-03 or vehicle (DMSO) and incubated overnight. Cells were collected and washed three times with PBS. To obtain cell lysates, the cells were suspended in 1 ml cold whole cell lysis buffer (50 mM Tris-HCl, pH 7.5, 0.5 M NaCl, 1% NP-40, 0.1% SDS) with protease and phosphatase inhibitors (P044-1ML, Sigma Aldrich). The cytosolic protein fraction was isolated by centrifugation at 13,000xg for 30 min at 4 °C to remove cellular debris. An aliquot of cell lysate normalized to cell number was mixed with 4-fold volume cold acetone to precipitate proteins. All samples were centrifuged at 13,000×g for 5 min. Protein precipitations were collected and evaporated to dryness on SpeedVac concentrator. The pellet was resuspended in sodium phosphate buffer (pH 8.0), reduced in 10 mM dithiothreitol (1 hr at 60°C), alkylated with 30 mM iodoacetamide (20 min, room temperature in the dark) and digested with 10 μg trypsin (Promega) at 37 °C on orbital shaker.

Tryptic peptides were purified by Pierce™ Graphite Spin Columns following manufacturer's instructions, resuspended in water with 0.1% formic acid (v/v) and analyzed by an LTQ Orbitrap XL mass spectrometer (Thermo Fisher Scientific) coupled to a Prominence Nano LC (Shimadzu) using the Xcalibur version 2.7.0 (Thermo Scientific). A total of 10 μL of sample was loaded and washed for 6 min on a C18-packed precolumn (1 cm × 150 μm, 5 μm, 200 Å, Michrom Bioresources, Auburn, CA) with a solvent of A:B=99:1 (A, 0.1% formic acid aqueous solution; B, 0.1% formic acid acetonitrile solution) at a constant flow of 12 μl/min. Peptides were transferred onward to a C18-packed analytical column (25 cm × 150 μm, 5 μm, 200 Å, Michrom Bioresources, Auburn, CA) and separated with a linear gradient of 6–55 min, 2–40% B, 55–62 min, 40–80% B, 62–70 min, 80% B (v/v) at the flow rate of 600 nl/min. The mass spectrometer performed a full MS scan (*m/z* 300–2000) at a resolution of 30000. The spray voltage, capillary temperature and capillary voltage were set to 2.0 kV, 200 °C, and 39.5 V, respectively. The three most intense ions were selected for fragmentation using collision-induced dissociation (CID) in the LTQ (normalized collision energy of 35, parent mass selection window of 2.5 Da, activation time of 30 ms, and minimum signal threshold for MS/MS scans set to 500 counts). Charge state rejection (charge state 1 was rejected) as well as dynamic exclusion (repeat counts, 2; repeat duration, 10 s; exclusion duration, 10 s) was enabled.

LC-MS/MS raw data were searched by Proteome Discoverer 1.4 using Sequest search engine (Thermo Fisher Scientific), against the Uniprot Human database (6/10/2016, 135431 sequences) at a false discovery cut off ≤1%. A maximum of two missed cleavage sites was allowed. The mass tolerance for the precursor ion was set on 10 ppm and for the fragment on 0.1 Da. Oxidation of methionine and phosphorylation of serine, threonine and tyrosine were enabled as dynamic modifications, while carbamidometylation of cysteine was set as fixed modification. The label-free quantification of peptides eluting between 10 and 80 min was performed with SIEVE 2.1 software (Thermo Scientific). Briefly, the chromatographic peaks detected by Orbitrap were aligned and the peptides peaks were detected with a minimum signal intensity of 1 × 10^5^; quantitative frames were determined based on *m/z* (width: 10 ppm) and retention time (width: 2.5 min). Statistical filters were set to assess the quality of the data. A *p*-value cutoff of 0.05 was used to define the altered proteins, and the CV raw MS intensities of the triplicates had to be within 30%. This helped minimize the effect of run-to-run variability. A list of differentially-expressed proteins and their gene/protein ID numbers was uploaded to the Ingenuity Pathway Analysis (IPA, Ingenuity Systems, CA) software for a Core analysis to investigate the protein function and biological networks.

### Statistical analysis

All graphs were prepared using GraphPad prism 6 software. Data are presented as mean ± SD or standard error of the mean (SEM) as indicated in the figure legends. Means were compared with Student t tests.

## SUPPLEMENTARY MATERIALS FIGURES AND TABLES



## Abbreviations

HIV-1: human immunodeficiency virus
PP1: protein phosphatase-1
DPs: degradation products
LC/FT-MS: liquid chromatography/Fourier transform mass spectrometry
SPR: surface plasmon resonance
PK: pharmacokinetic
i.p.: intraperitoneal
