# Supplementary material for: Inhibition of HIV-1 infection in humanized mice and metabolic stability of protein phosphatase-1-targeting small molecule 1E7-03
Source: Oncotarget. 2017 Aug 7;8(44):76749–69. doi: 10.18632/oncotarget.19999 (PMC5652740; doi:10.18632/oncotarget.19999)
Supplement: Supplementary file 1 [file oncotarget-08-76749-s001.pdf]

Inhibition of HIV-1 infection in humanized mice and metabolic stability of protein phosphatase-1-targeting small molecule 1E7-03

SUPPLEMENTARY MATERIALS

Analysis of 1E7-03 fragmentation pattern by mass spectrometry

To obtain the optimal settings for spray voltage, capillary temperature, capillary voltage and tube lens offset voltage, 1E7-03 (100 mM in acetonitrile) was injected using a syringe pump into the mass spectrometer at a flow rate of 3 μl/min. The optimized parameters were applied to the analysis of 1E7-03, which included collection of MS^3 spectra and fragmentation patterns (Supplementary Figure 6 and Supplementary Table 1). The positive ion mode (+)-ESI-MS^n spectra of 1E7-03 identified a precursor ion with *m/z* =504.2138 (Supplementary Figure 6, MS^1 panel), which was further cleaved to form two major fragment ions with *m/z* =433.1771 and *m/z* =459.1563 (Supplementary Figure 6, MS^2 panel). The most abundant MS^2 fragment ion with *m/z*=433.1771 was formed by the apparent loss of the isocyanatoethane moiety (Supplementary Figure 6, see a diagram on the right). The MS^3 analysis showed the formation of a product ion with *m/z* =362.1394 (Supplementary Figure 6, MS^3 panels). This product ion was likely formed by the loss of the acrylamide moiety (Supplementary Figure 6, see a diagram on the right). Elimination of ethylamine moiety from the parent ion with *m/z*= 504.2138 resulted in the formation of another MS^2 fragment ion with *m/z* =459.1563 (Supplementary Figure 6, MS^2 panel), which was also cleaved in the MS^3 analysis into the product ion with *m/z* =362.1392 (Supplementary Figure 6, MS^3 panel). This ion was formed through the loss of 2-propenoyl isocyanate moiety (Supplementary Figure 6, see a diagram on the right). The fragmentation pattern of 1E7-03 was further supported by the mass spectra data and fragmentation pattern of its analog (compound 7d (Ammosova et al., 2014)) (Supplementary Figure 7 and Supplementary Table 2). Taken together, the fragmentation analysis showed formation of three major ions resulted from the breakdown of the parental compound 1E7-03.

LC/FT-MS instrument validation

The synthesized compounds were used for validation of LC/FT-MS procedure. The elution time for 1E7-03, DP1 and DP3 were determined to be 34.89 ± 0.03 min, 33.63 ± 0.03 min, 27.93 ± 0.03 min (n = 5) (Supplementary Figure 8A). All three compounds were detected with a good linearity within the concentration range of 0.1-5 mM for 1E7-03 and 0.1-10 mM for DP1 and DP3 with an r^2 value of 0.9968-1.0000 (Supplementary Figure 8B-8D). The limit of detection (LOD) and limit of quantification (LOQ) values were found to be 1.20-5.66 nM and 3.99-18.87 nM respectively (Supplementary Table 3). The intraday (n = 5) and inter-day (n = 5) precision of the method was good with a relative standard deviation (RSD) lower than 5% (Supplementary Table 4). The percent mean recoveries of 1E7-03, DP1 and DP3 were within the acceptable range of 93.05-108.36% (Supplementary Table 5). Minor variations of chromatographic conditions did not affect the resolution of 1E7-03 and its DPs, demonstrating good robustness of the method. Thus the developed method was suitable for the analysis of 1E7-03 degradation and was further applied to determine the degradation kinetics of 1E7-03 in biological fluids.

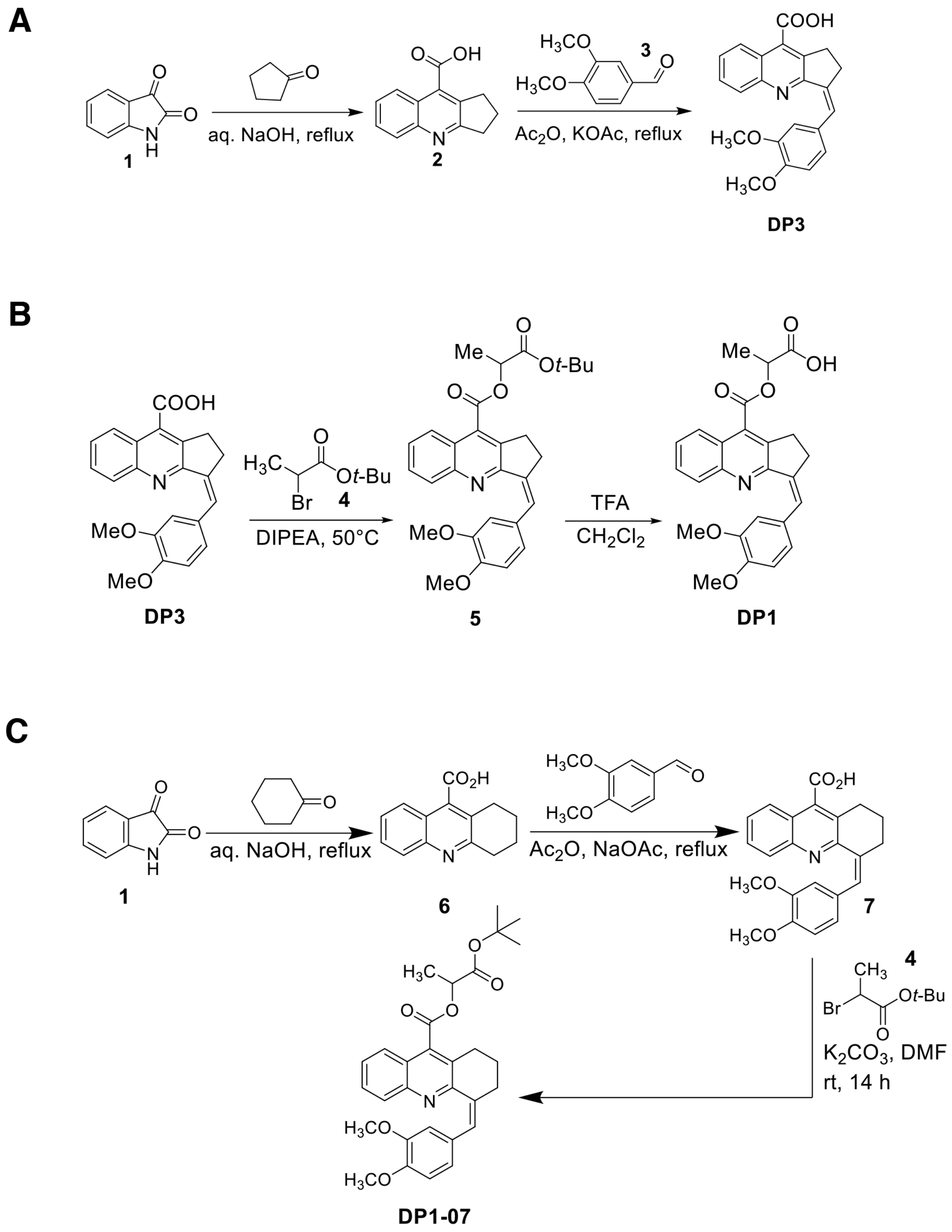

Supplementary Figure 1: Chemical synthesis of 1E7-03 degradation products. **(A)** DP3, **(B)** DP1 and **(C)** DP1-07.

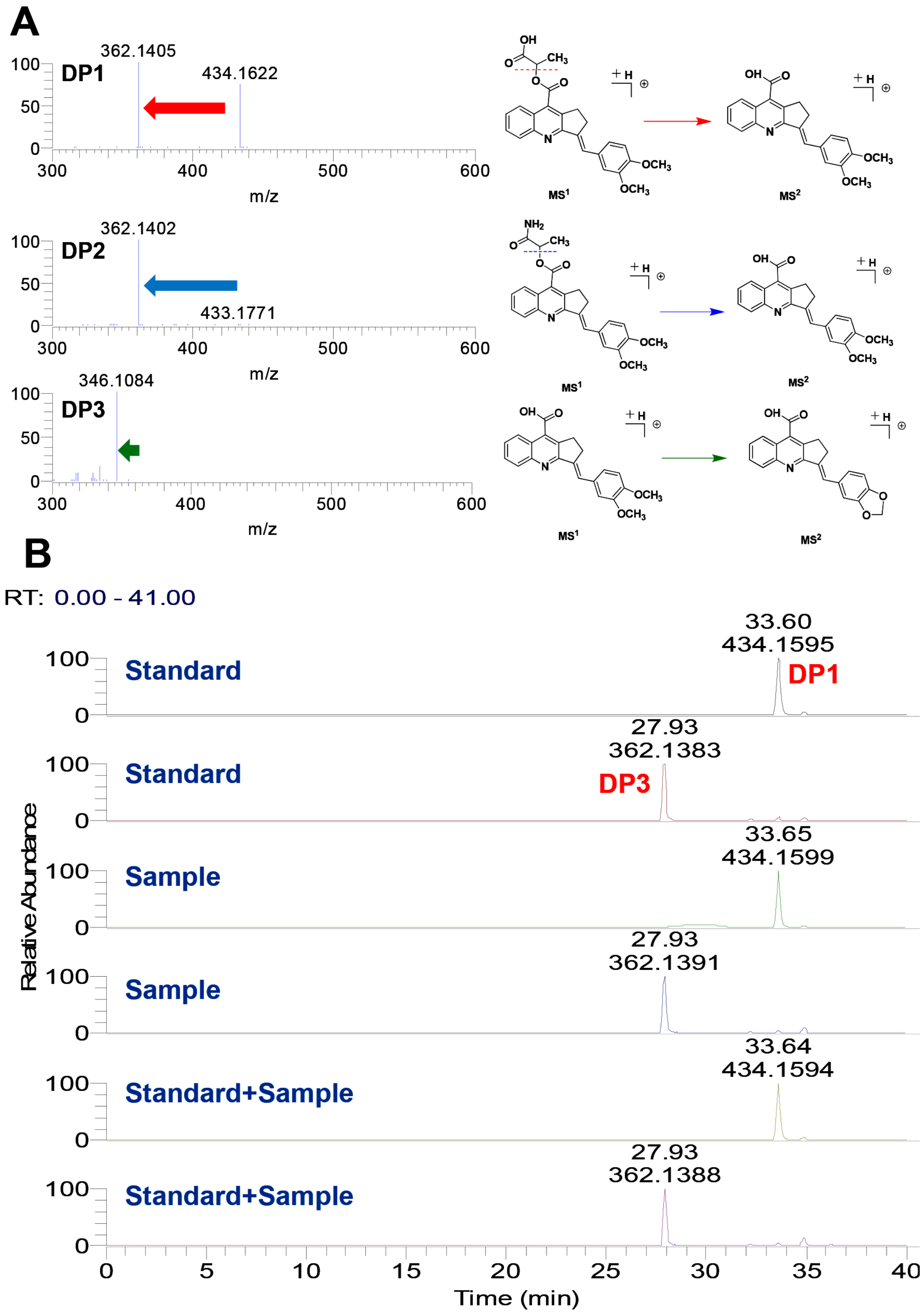

Supplementary Figure 2: Structural characterization of DP1, DP2 and DP3. (A) MS^2 spectra and fragmentation patterns of DP1, DP2 and DP3. **(B)** Comparing retention times and MS data of DP1 and DP3 in samples with their synthesized references.

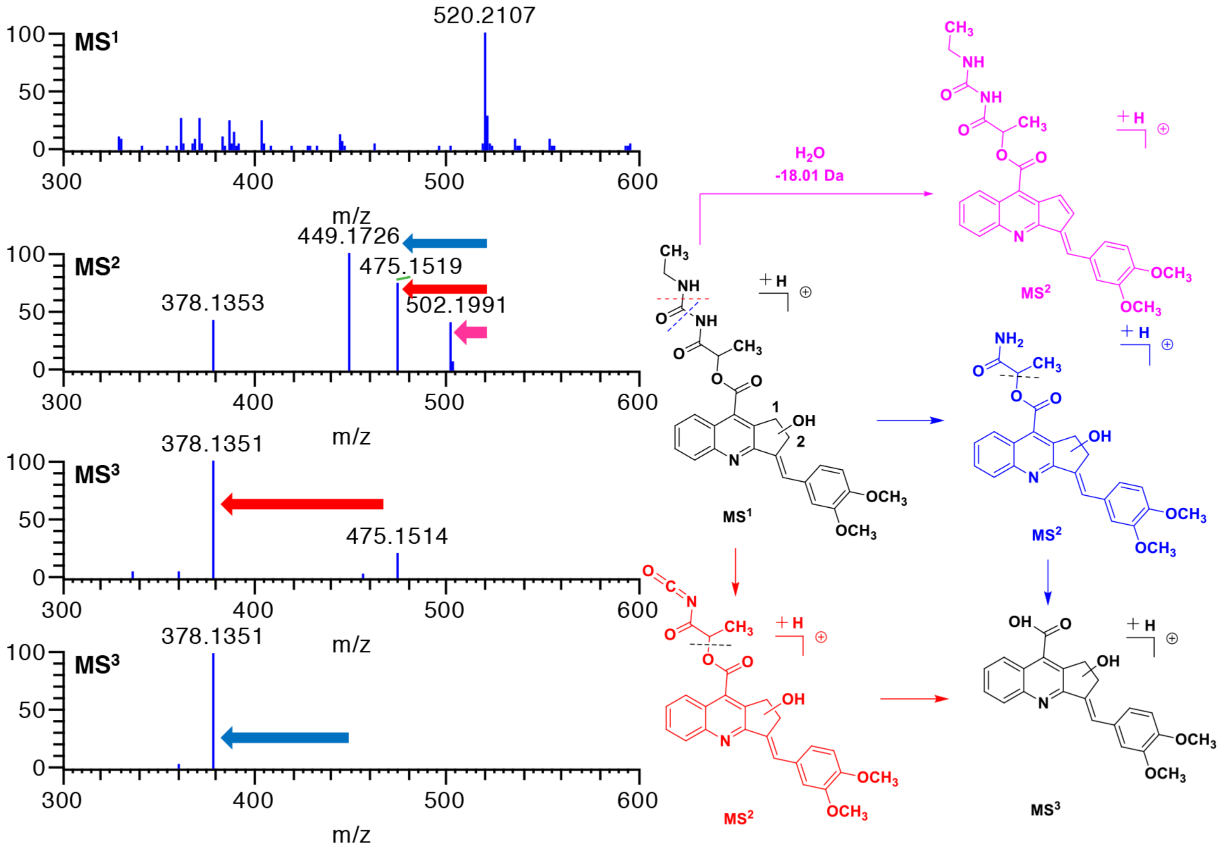

Supplementary Figure 3: Structural characterization of DP4 (hydroxylation on the 2, 3-dihydro-1H-cyclopenta ring). MS^1, the precursor ion *m/z*=520.2107 of DP4. MS^2, three product ions of *m/z*=502.1991, *m/z*=475.1519, *m/z*=449.1726 derived from the precursor ion of *m/z*=520.2107. MS^3, the product ion with *m/z* =378.1351 was generated by further cleavage of ions with *m/z* =475.1519 and *m/z* =449.1726.

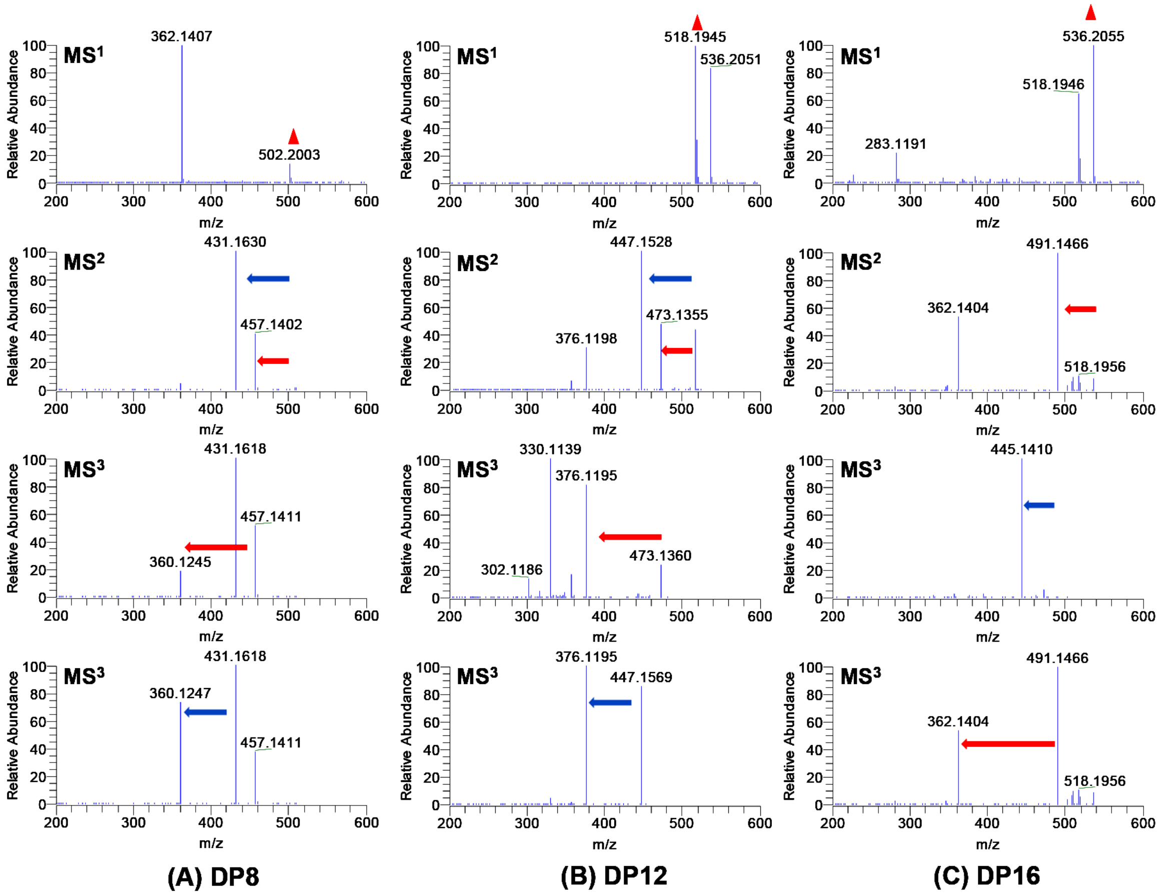

Supplementary Figure 4: The MS/MS spectra of DP8, DP12 and DP16 (hydroxylation on the 2,3-dihydro-1H-cyclopenta ring). The high-accuracy molecular weights of DP8, DP12 and DP16 observed in the FT-MS spectra were *m/z* 502.1992, 518.1950 and 536.2057, corresponding to the elemental composition of $[C_{28}H_{28}N_3O_6]^+$, $[C_{28}H_{28}N_3O_7]^+$ and $[C_{28}H_{30}N_3O_8]^+$, respectively. It is suggested that DP8/DP12 could be the dehydrogenation products of 1E7-03/DP4, and DP16 could be the dihydroxylation product of 1E7-03.

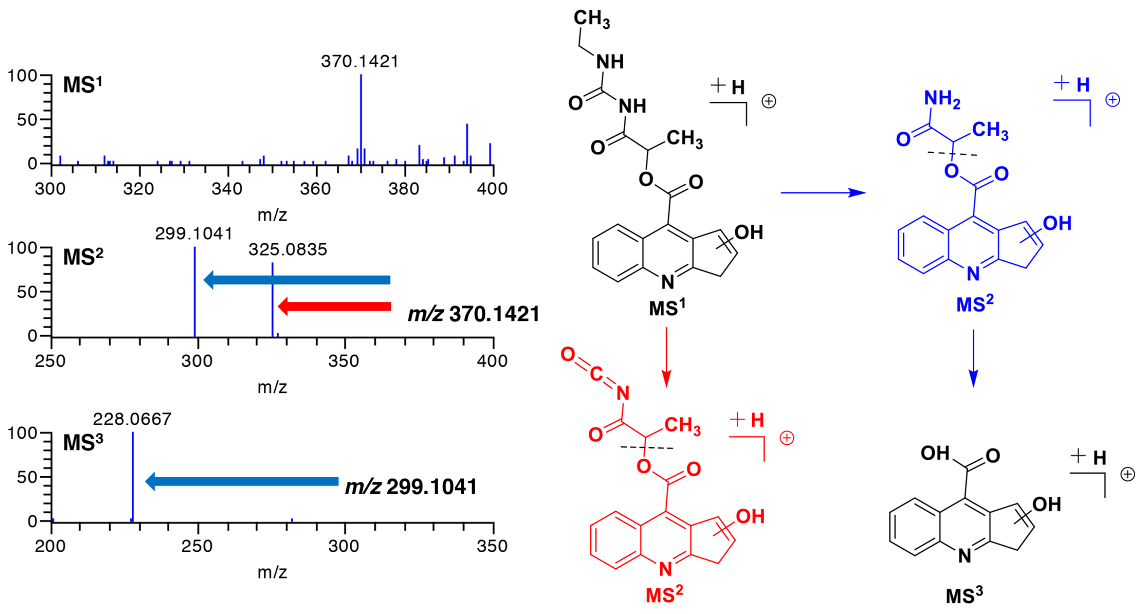

Supplementary Figure 5: Structural characterization of DP18 (the loss of 3-(3,4-dimethoxyphenyl) methylene). MS^1, the precursor ion *m/z*=370.1421 of DP18. MS^2, cleavage of the precursor ion *m/z* =370.1421 into two MS^2 fragment ions with *m/z* =325.0835 and *m/z* =299.1041. MS^3, further cleavage of ion with *m/z* =299.1041 into the product ion with *m/z* =228.0667.

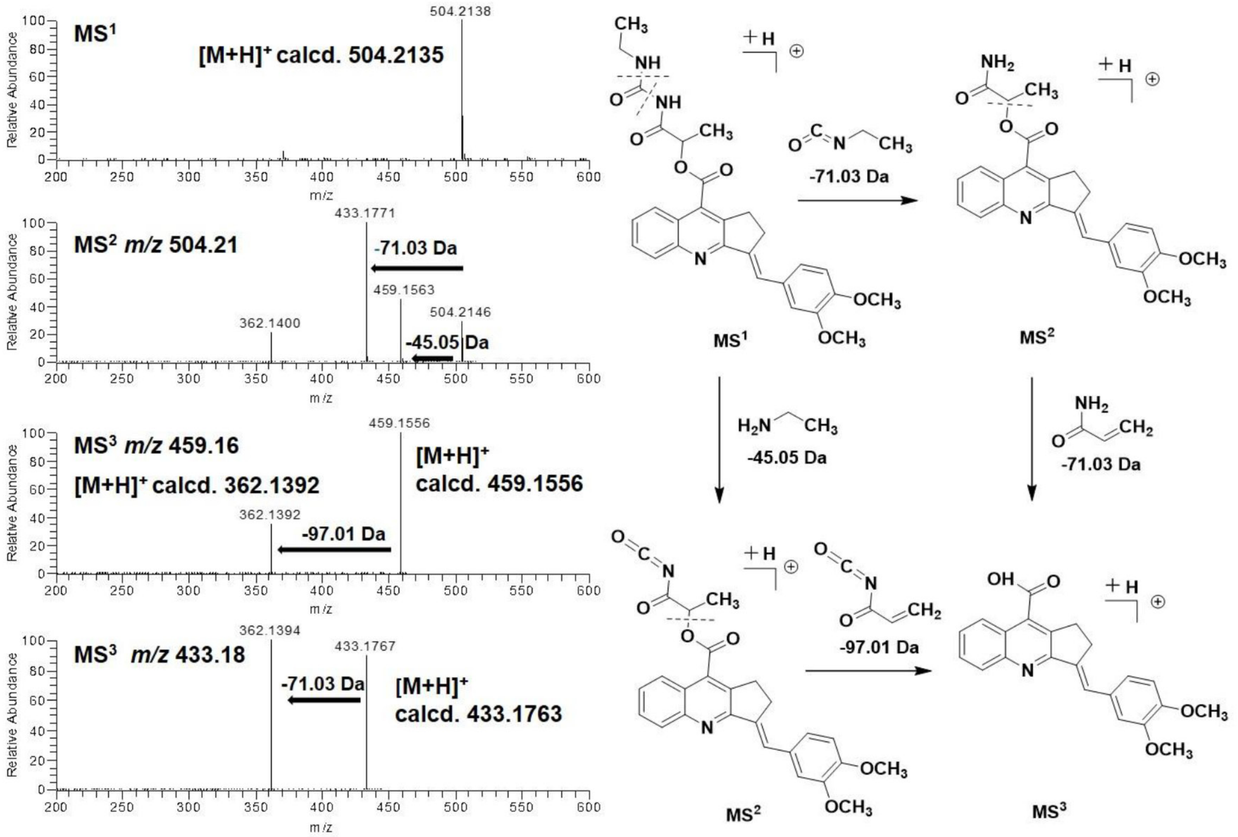

Supplementary Figure 6: Fragmentation pattern of 1E7-03. (+) ESI-MS^n spectra and fragmentation pattern of 1E7-03 are shown. MS^1, the precursor ion *m/z* =504.2138 of 1E7-03. MS^2, cleavage of the precursor ion *m/z* =504.2138 into two MS^2 fragment ions with *m/z* =433.1771 and *m/z* =459.1563. MS^3, further cleavage of ions with *m/z* =433.1771 and *m/z* =459.1563 into the product ion with *m/z* =362.1392.

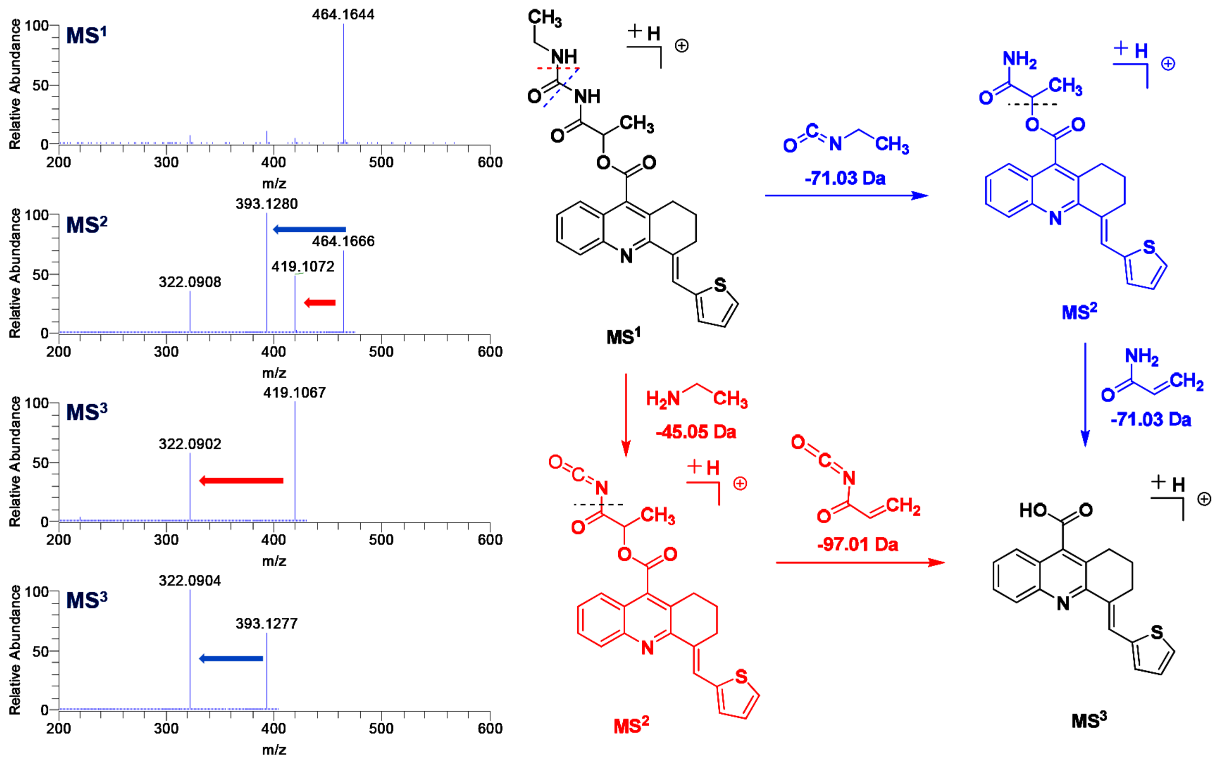

Supplementary Figure 7: MS^n spectra and fragmentation patterns of compound 7d. MS^1, the precursor ion *m/z* =464.1644 of compound 7d. MS^2, cleavage of the precursor ion *m/z* =464.1644 into two MS^2 fragment ions with *m/z* =419.1072 and *m/z* =393.1280. MS^3, further cleavage of ions with *m/z* =419.1072 and *m/z* =393.1280 into the product ion with *m/z* =322.0902.

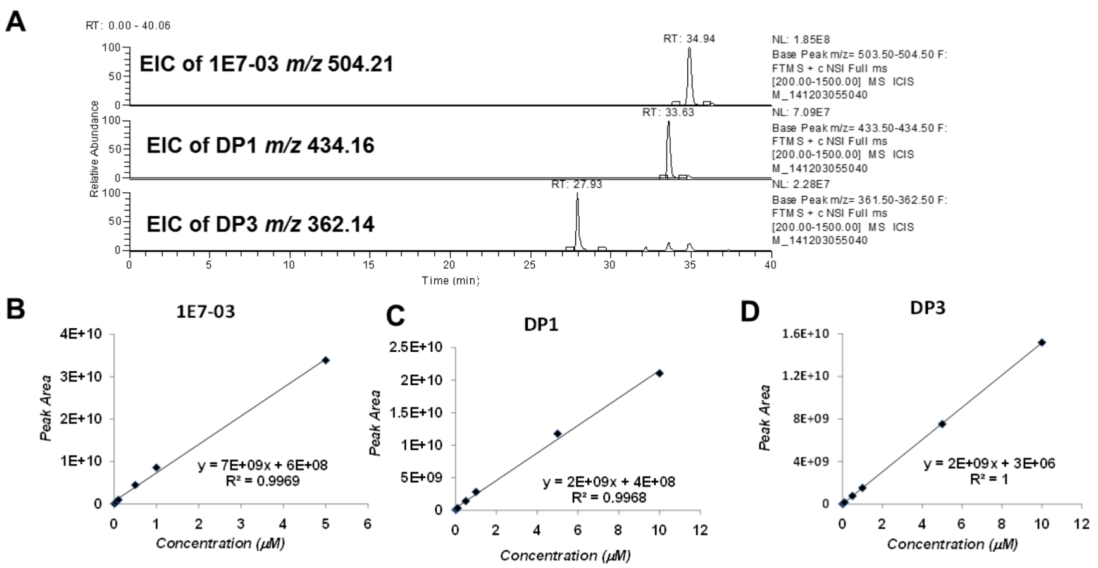

Supplementary Figure 8: Validation of LC/FT-MS procedure for 1E7-03 analysis. **(A)** Representative extract ion chromatogram (EIC) spectra of 1E7-03, DP1 and DP3 obtained during LC/FT-MS analysis. Calibration plots for **(B)** 1E7-03, **(C)** DP1 and **(D)** DP3 obtained with LC/FT-MS.

Supplementary Table 1: High-resolution mass spectral data of 1E7-03 by (+)-ESI-FT-MS

MS^n	Ions (*m/z*)	Pred. (*m/z*)	Diff. (mDa)	Diff. (ppm)	Formula
MS^1	504.2138	504.2135	0.30	0.59	$C_{28}H_{30}N_3O_6^+$
MS^2	459.1563	459.1556	0.70	1.52	$C_{26}H_{23}N_2O_6^+$
MS^2	433.1771	433.1763	0.80	1.85	$C_{25}H_{25}N_2O_5^+$
MS^3	362.1394	362.1392	0.20	0.55	$C_{22}H_{20}N_1O_4^+$
MS^3	362.1392	362.1392	0.00	0.00	$C_{22}H_{20}N_1O_4^+$

Supplementary Table 2: High-resolution mass spectral data of compound 7d (an analog of 1E7-03) by (+)-ESI–FT-MS

MS^n	Ions (*m/z*)	Pred. (*m/z*)	Diff. (mDa)	Diff. (ppm)	Formula
MS^1	464.1644	464.1644	0.00	0.00	$C_{25}H_{26}N_3O_4S^+$
MS^2	419.1072	419.1066	0.60	1.43	$C_{23}H_{19}N_2O_4S^+$
MS^2	393.1280	393.1273	0.70	1.78	$C_{22}H_{21}N_2O_3S^+$
MS^3	322.0902	322.0902	0.00	0.00	$C_{19}H_{16}NO_2S^+$
MS^3	322.0904	322.0902	0.20	0.62	$C_{19}H_{16}NO_2S^+$

Supplementary Table 3: Calibration curves of 1E7-03, DP1 and DP3

Analyte	Calibration curve	r^2	Linear range (mM)	LOD (nM)	LOQ (nM)
**1E7-03**	$y = 7\times10^9 x + 6\times10^8$	0.9969	0.01–5	5.66	18.87
**DP1**	$y = 2\times10^9 x + 4\times10^8$	0.9968	0.01–10	1.20	3.99
**DP3**	$y = 2\times10^9 x + 3\times10^6$	1.0000	0.01–10	4.98	16.59

Note: y, peak area; x, concentration of each compound (μM); LOD, limit of detection (Signal/Noise=3); LOQ, limit of quantification (Signal/Noise=10).

Supplementary Table 4: The intra-day (n = 5) and inter-day (n = 5) precision of the method

Analyte		Intra-day						
		1	2	3	4	5	Average	RSD%
**1E7-03**	Area	985766965	956783317	970859056	983338943	989616679	977272992	1.23
**DP1**	Area	11945674886	11945674886	11945674886	11945674886	11945674886	11772532600	0.72
**DP3**	Area	1508770552	1497723766	1532452199	1550238740	1516960262	1521229104	1.35
**Analyte**		**Inter-day**						
		**1**	**2**	**3**	**4**	**5**	**Average**	**RSD%**
**1E7-03**	Area	7906876990	7346269055	7449367838	7862922255	7777333868	7668554001	3.31
**DP1**	Area	2761941309	2902489486	2856726475	2849617981	2795750432	2833305137	1.94
**DP3**	Area	1841726496	1807220482	1856866952	1927090727	1919800681	1870541068	2.76

Supplementary Table 5: Recoveries of the method

Analyte	Matrice	Conc. Spiked (ng/mL)	Conc. Calcd. (ng/mL)	Recovery (%)
	Serum	121.19	123.51	101.91
**1E7-03**	Medium	121.19	127.84	105.48
	Buffer (PH=7)	121.19	122.49	101.08
	Serum	65.51	70.98	108.36
**DP1**	Medium	65.51	60.96	93.05
	Buffer (PH=7)	65.51	68.56	104.65
	Serum	53.27	57.31	107.58
**DP3**	Medium	53.27	54.54	102.40
	Buffer (PH=7)	53.27	49.73	93.37

Note: recovery (%) = concentration calculated/concentration spiked × 100%.
